# Investigation of the Therapeutic Effect of Total Alkaloids of *Corydalis saxicola* Bunting on CCl_4_-Induced Liver Fibrosis in Rats by LC/MS-Based Metabolomics Analysis and Network Pharmacology

**DOI:** 10.3390/metabo13010009

**Published:** 2022-12-21

**Authors:** Qianyi Wang, Zhuo Luo, Danfeng Li, Jinghua Qin, Ziping Pan, Bingjian Guo, Lijun Deng, Yunyuan Nong, Zheng Huang, Ying He, Hongwei Guo, Dan Zhu, Yonghong Liang, Zhiheng Su

**Affiliations:** 1 Pharmaceutical College, Guangxi Medical University, No. 22 Shuang Yong Road, Qingxiu District, Naning 530021, China; 2 Guangxi Institute for Food and Drug Control, Nanning 530021, China; 3 Key Laboratory of Longevity and Aging-Related Diseases of Chinese Ministry of Education & Center for Translational Medicine, Guangxi Medical University, Nanning 530021, China; 4 Guangxi Beibu Gulf Marine Biomedicine Precision Development and High-Value Utilization Engineering Research Center, Guangxi Medical University, No. 22 Shuang Yong Road, Qingxiu District, Naning 530021, China; 5 Guangxi Health Commission Key Laboratory of Basic Research on Antigeriatric Drug, Guangxi Medical University, No. 22 Shuang Yong Road, Qingxiu District, Naning 530021, China; 6 Guangxi Key Laboratory of Bioactive Molecules Research and Evaluation, Guangxi Medical University, No. 22 Shuang Yong Road, Qingxiu District, Naning 530021, China

**Keywords:** total alkaloids of *Corydalis saxicola* Bunting, liver fibrosis, metabolomics, UPLC-Q-TOF/MS, network pharmacology

## Abstract

Liver fibrosis is a pathological result of liver injury that usually leads to a pathophysiological wound healing response. The total alkaloids of *Corydalis saxicola* Bunting (TACS) have been used for hepatoprotective effects on the liver. However, its exact therapeutic mechanisms of liver fibrosis are not yet well understood. To explore the potential anti-fibrosis mechanism of TACS, metabolomics coupled with network pharmacology were applied to reveal the underlying mechanisms. Ultra-performance liquid chromatography quadrupole time-of-flight mass spectrometry (UPLC-Q-TOF/MS) combined with multivariate statistical analyses were performed to estimate changes in metabolic profiles. As a result, a total of 23 metabolites in rats with liver fibrosis were altered; of these, 11 had been downregulated and 12 had been upregulated compared with the control group. After TACS treatment, the levels of 13 metabolites were significantly restored compared with the CCl_4_-treated group, of which 4 metabolites were up-regulated and 9 metabolites were down-regulated. Many of these metabolites are involved in the bile acid metabolism, glutathione metabolism, tryptophan metabolism and purine metabolism. Then, three key targets, including cytochrome P450 family1 subfamily A member 1 (CYP1A1), ornithine decarboxylase 1 (OCD1) and monoamine oxidase Type B (MAOB) were predicted as potential therapeutic targets of TACS against liver fibrosis through network pharmacology analysis. Finally, palmatine, tetrahydropalmatine and dehydrocavidine were screened as potential active compounds responsible for the anti-fibrosis effect of TACS by molecular docking analysis. This study reveals that TACS exerted anti-fibrosis effects by regulating the liver metabolic pathway with multiple components and multiple targets, which is helpful to further clarify the hepatoprotective mechanisms of natural plant extracts.

## 1. Introduction

Chronic liver diseases are a major global health burden, causing approximately 2 million deaths worldwide each year [1]. Liver fibrosis is an active repair reaction of chronic liver injury caused by various pathogenic factors. It is a major pathological factor causing massive deposition of the extracellular matrix (ECM) that can destroy the normal structure of liver, eventually leading to cirrhosis and hepatocellular carcinoma (HCC) [2,3]. Therefore, the prevention and treatment of liver fibrosis is particularly critical. However, to date, there are no specific effective drugs available for liver fibrosis treatment in the clinical setting. Accumulating evidences have demonstrated that Chinese herbal medicine has good preventive and therapeutic effects in various liver diseases, such as liver injury [4], liver fibrosis [5], hepatitis and cirrhosis [6], and has some unique advantages including high availability, multiple targets and few side effects.

*Corydalis saxicola* Bunting (CS) is a plant of the genus *Corydalis* (Papaveraceae), a light green perennial herb that grows in mountain rock crevices and is majorly distributed in southern China [7]. Total alkaloids of *Corydalis saxicola* Bunting (TACS) are the main active components derived from CS, a natural plant extracts that has been historically used to cure inflammation, tumors and hepatic diseases [8]. A growing amount of evidence has demonstrated that CS and its alkaloids can be use as hepatoprotective agents. Zeng et al. found that dehydrocheilanthifoline could effectively inhibit the replication of hepatitis B virus [9]. An in vivo experiment showed that dehydrocavidine can alleviate liver fibrosis by regulating oxidative stress and the expression of fibrosis-related genes (Bcl2, Cyp3a13, IL18 and Rad50) [10]. Moreover, our previous studies have explored the protective effect of CS on both acute hepatotoxicity and liver fibrosis using a CCl_4_-induced rat model, which showed that CS could synergistically regulate amino acid metabolism, glucose metabolism and lipid metabolism [5,11]. Wu et al. found that TACS can alleviate liver injury by regulating the disorder of intestinal bacteria metabolism and amino acid metabolism [4]. However, the mechanism of TACS in treating liver fibrosis has not yet been clarified.

Metabolomics and network pharmacology are two burgeoning disciplines linking metabolites to disease targets to reveal the mechanism of drug action. Metabolomics as a new method was used to characterize small metabolites (a molecular weight of less than 1000) in biological fluids or tissues by using advanced high-throughput analytical chemistry techniques [12]. This strategy has been extensively applied to explore the effect of natural plant extracts on liver diseases. Yao et al. used liver metabolomics to explore the liver-protecting effect of steamed shoot extracts of ginseng (*Panax ginseng* C.A. Meyer) [13]. The fecal metabolomics analysis showed that *Phyllanthus emblica* extracts restored the disturbed metabolites in NAFLD mice [14]. Therefore, it is feasible to evaluate the protective effect of natural extracts on liver diseases and detect the change in related metabolites with metabolomics analysis. Liquid chromatography-mass spectrometry (LC-MS) is one of the most commonly used analytical tools in metabolomics studies with high sensitivity and specificity [15,16]. Natural plant extracts contain many components that can interfere with the related targets of the metabolic network [17]. Network pharmacology is a comprehensive analysis approach, which can reveal the synergistic effect between multiple components with multiple targets and help to systematically understand the therapeutic mechanism of natural plant extracts [18].

In this study, fecal metabolomics and network pharmacology were applied to investigate the protective mechanism of TACS for liver fibrosis. In combination with multivariate statistical analysis, the potential biomarkers and related pathways of TACS treatment for CCl_4_-induced liver fibrosis were further revealed. This research aims to assess the potential of TACS in anti-liver fibrosis and provide a new perspective for the exploration of the liver-protective effects of natural plant extracts.

## 2. Material and Methods

### 2.1. Drugs and Chemicals

Superoxide dismutase (SOD) and Malondialdehyde (MDA) were purchased from Nanjing Jiancheng Bioengineering Institute (Nanjing, China). CCl_4_ solution was purchased from Adamas (Shanghai, China). Colchicine was purchased from Xishuangbanna Pharmaceutical Co., Ltd. (Jinghong, China). Olive oil was purchased from Macklin (Shanghai, China).

### 2.2. Preparation of TACS

The *C. saxicola* Bunting herbs were obtained from Donglan County, Hechi City, Guangxi Province, which was authenticated by Professor Dan Zhu of Guangxi medical university. The voucher specimen (CS-HCDL-20201015) was deposited at the Museum of Traditional Chinese Medicine Specimens of Guangxi Medical University (Nanning, China). UPLC-Q-TOF/MS was applied to the TACS qualitative analysis. The results are provided in the Supplementary Materials.

### 2.3. Animals and Treatments

Thirty-two male Sprague Dawley rats (180–220 g) were purchased from the Changsha Tianqin Biotechnology Co., LTD (Changsha, China) Approval No. SYXK(GUI)2014-0002. The animal experiment of this study received animal welfare ethics approval by Guangxi Medical University: No:202112005. All rats were raised in a controlled-temperature (20–25 °C) environment (12 h light/dark cycle) under relative humidity (40–60%). All animals were randomly divided into 4 groups (*n* = 8), including: control, CCl_4_-treated, TACS and colchicine (COL). The CCl_4_-treated, TACS and COL groups received CCl_4_ (0.1 mL/100 g, i.g) and olive oil mixed at the rate of 1:1 (*v*/*v*) twice a week for 10 weeks, and the control group was administered with the same dose of soybean oil.

**Figure 1:**
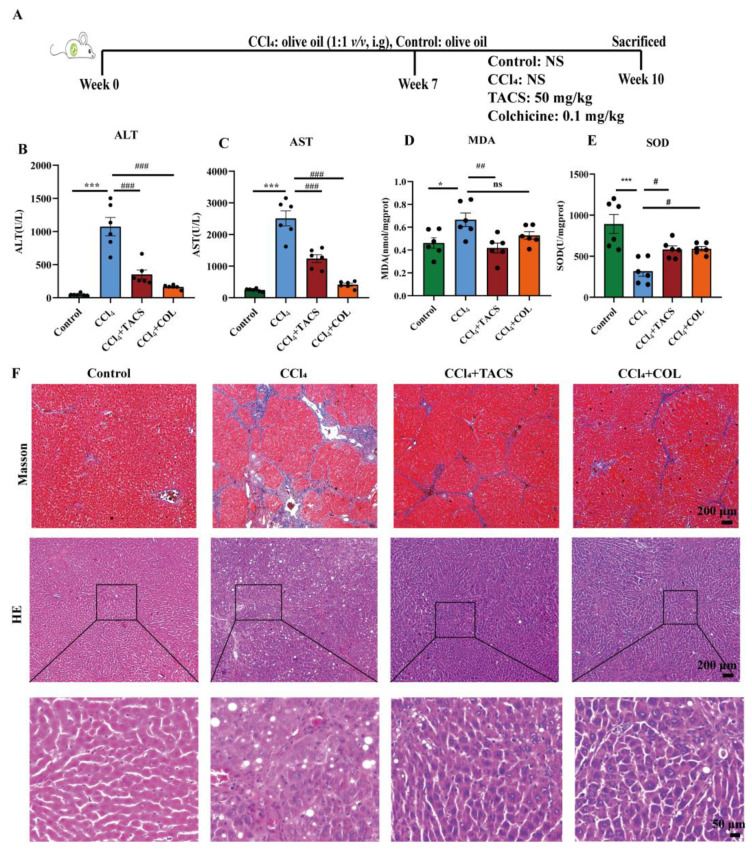
Effects of TACS on liver fibrosis in CCl_4_-induced rats. (**A**) Animal experiment design (**B**,**C**) The serum of ALT and AST levels (*n* = 6). (**D**,**E**) The liver of MDA and SOD levels (*n* = 6). (**F**) Representative Masson and H&E staining of liver tissue photos. Data were expressed as the mean ± SEM. * *p* < 0.05, *** *p* < 0.001 vs. control group; ^#^ *p* < 0.05, ^##^ *p* < 0.01, ^###^ *p* < 0.001 vs. CCl_4_-treated group, ns represents no significance.

Since the 7th week, the rats in the TACS group were treated with a dose of 50 mg/kg body weight of TACS (0.5 mL/100 g, ig) and the COL group were treated with a dose of 0.1 mg/kg body weight of colchicine (0.5 mL/100 g, ig) once daily for 4 weeks, other rats were administered with the same dose of normal saline (Figure 1A).

### 2.4. Sample Collections and Preparation

All rats were sacrificed 24 h after the last drug administration, and blood samples were collected by puncturing the abdominal aorta. Serum was isolated by centrifugation (1200 × *g*, for 10 min). The liver samples were taken from the same region and were fixed in 4% neutral formalin. The remaining area of the liver was stored at −80 °C for future use.

The fecal samples were processed with ice water (500 μL/100 mg), vortexed, blended and centrifuged (16,200 × *g*, 4 °C, for 15 min). Then, the researchers collected the supernatant and further processed the samples with ice methanol in the same way. The double extraction mixture was centrifuged (16,200 × *g*, 4 °C, for 20 min) [19].

### 2.5. Biochemical Analysis

Activities of alanine aminotransferase (ALT) and aspartate aminotransferase (AST) were determined using a HITACHI automatic biochemical analyzer 7100 (Hitachi Ltd., Tokyo, Japan). The liver malondialdehyde (MDA) and superoxide dismutase (SOD) were measured according to the manufacturer’s instructions.

### 2.6. Histopathological Assessment

Liver samples were fixed in 4% buffered formalin and embedded in paraffin. Liver histopathologic changes were examined using Masson and H&E staining under a light microscope (MT52-N, Guangzhou, China).

### 2.7. Immunohistochemistry (IHC)

Immunohistochemistry (IHC) was used for the detection of alpha-smooth muscle actin (α-SMA) and Collagen Type I (collagen-I) in liver tissue sections. After deparaffinization, rehydration, antigen unmasking by heat treatment, and 5% BSA blocking, sections were incubated with anti-α-SMA (1:2500; 67735-1-Ig, proteintech) and anti-collagen-I (1:2500; 67735-1-Ig, proteintech) overnight at 4 °C. After rinsing with PBS buffer solution 3 times, the tissue sections were incubated with the secondary antibody of the goat anti-mouse at room temperature (for 1 h). Antigens were marked by DAB reaction mixture, followed by staining with hematoxylin. Then, sections were observed under a microscope (MT52-N, Guangzhou, China). Image-J software was used to measure positively stained areas.

### 2.8. Metabolites and Metabolic Pathway Analysis

#### 2.8.1. UPLC-Q-TOF/MS Analysis

The fecal extracts were separated by Waters ACQUITY UPLC system (Waters Corp., Milford, MA, USA) on an ACQUITY UPLC BEH C18 column (100 × 2.1 mm, 1.7 µm). For the fecal sample analysis, mobile phases A and B were water/0.1% formic acid and acetonitrile with a 0.30 mL/min flow rate and columns was 40 °C. The flow was programmed to start with 98% A and 2% B, 0 to 3.0 min; 75% A and 25% B, 3.0 to 10.0 min; 60% A and 40% B, 10.0 to 15.0 min; 30% A and 70% B, 15.0 to 18.0 min; 10% A and 90% B, 18 to 21 min; 98% A and 2% B, 21 to 25 min. The autosampler was maintained at 4 ℃ and injection volume was 5 µL.

Mass analysis with an electrospray ionization source operating in positive ion mode was performed on a XEVO G2-XS QTOF mass spectrometer (Waters Corp., Manchester, UK). The conditions were set as follows: the capillary voltage, 3.0 kV; the sample voltage, 40 V, extraction cone voltage, 4.0 V. The desolvation gas rate was 800 L/h and temperature was set at 400 °C; the cone gas rate was 40 L/h; the source temperature was 100 °C; and the scan time and interscan delay were 0.15 s and 0.02 s, respectively. Leucine-enkephalin was used for the lock mass in all analyses ([M + H]^+^ 556.2771).

#### 2.8.2. Method Validation

Quality control (QC) samples were prepared by mixing equal volumes of fecal samples. The repeatability was evaluated by injecting QC samples nine times and extracting 8 ions. To evaluate the stability of the system, QC samples were analyzed every five samples. The results are shown in Table S2.

#### 2.8.3. Data Processing and Multivariate Analysis

The raw data were preprocessed by Marker Lynx Applications Manager version 4.1 (Waters, Manchester, UK), which includes integration, normalization peak and alignment. A list of intensities, including retention time and m/z data pairs were imported into SIMCA-P 14.1 software (Umetrics, Umeå, Sweden) for multivariate pattern recognition analysis, which includes principal component analysis (PCA) and partial least squares (PLS-DA) and orthogonal partial least squares discriminant analysis (OPLS-DA). The S-plot was used to observe the variables that contributed to the classification. The key variables were screened based on the variable importance in projection (VIP) values > 1 and *p*-values < 0.05 in order to assess the predictive abilities of the constructed OPLS-DA model by seven-fold cross-validation.

The key metabolites were identified by the *m/z* with HMDB (http://www.hmdb.ca) (accessed on 2 July 2022), and MassBank (http://www.massbank.jp) (accessed on 12 July 2022). The comprehensive metabolic network map was based on Kyoto Encyclopedia of Genes and Genomes (KEGG) (https://www.genome.jp/kegg/pathway.htm) (accessed on 4 August 2022), and MetaboAnalyst5.0 (https://www.metaboanalyst.ca/) (accessed on 10 August 2022).

#### 2.8.4. Statistical Analysis

Statistical differential analyses using one-way ANOVA or the Wilcoxon rank-sum test were conducted using SPSS20.0 (SPSS Software USA), and the data are expressed as mean ± SEM. GraphPad Prism8 (GraphPad Software USA) was applied for data plotting. Values of *p* < 0.05 were considered statistically significant.

### 2.9. Network Pharmacology Analysis

First, the Swiss Target Prediction database (http://www.swisstargetprediction.ch/) (accessed on 5 September 2022) and Batman-TCM were applied to search for TACS-related targets. Secondly, liver fibrosis-related targets were searched from Gene Cards (https://www.genecards.org/) (accessed on 5 September 2022), OMIM (https://omim.org/) (accessed on 5 September 2022) databases and TTD (http://db.idrblab.net/ttd/) (accessed on 5 September 2022). Cytoscape3.7.2. was applied to construct the “compound-target” network map and protein interaction (PPI) map.

### 2.10. Molecular Docking

The 2D structures of the chemical composition of TACS were searched from PubChem Compound (https://www.ncbi.nlm.nih.gov/pccompound) (accessed on 10 September 2022), and then, the 2D structure was converted to mol2 format by using OpenBabel-2.4.1. The crystal structures of the targets were found in the RCSB Protein Data Bank (https://www.rcsb.org/) (accessed on 10 September 2022). Three key protein targets were studied: cytochrome P450 family1 subfamily A member 1 (CYP1A1, PDB ID:4I8V), ornithine decarboxylase1(ODC1, PDB ID: 1D7k), and monoamine oxidase Type B (MAOB, PDB ID: 1O5W). The molecular docking analysis was performed using SYBYL-X version 2.0. Finally, PyMoL2.5 was applied to visually assess the docking results with the highest scores.

## 3. Results

### 3.1. Effects of TACS on Liver Fibrosis in CCl_4_-Induced Rats

To explore whether TACS could alleviate liver fibrosis, serum levels of AST and ALT were determined. The serum levels of AST and ALT were increased by CCl_4_. However, TACS and COL treatment reversed the levels of AST and ALT in the serum significantly (Figure 1B,C). It was also found that the level of liver MDA and the activity of SOD were obviously reversed in the TACS and COL groups compared with the CCl_4_-treated group (Figure 1D,E).

Masson staining is a gold index used to evaluate the success of liver fibrosis models [20]. In this study, Masson staining showed that the structure of the hepatic lobule was destroyed and exhibited extensive hepatic fibrosis compared with the control group, indicating that the liver fibrosis model is successful. After being treated with TACS and COL, the hepatic fibrosis was clearly alleviated (Figure 1F). H&E staining was applied to evaluate the morphology and structure of the liver. The hepatic lobule and hepatic cell were destroyed, and significant inflammatory infiltration and vacuole-like lesions were observed in the CCl_4_-treated group (Figure 1F). TACS and COL treatment alleviated these pathological changes. The results showed that TACS has protective effects in liver fibrosis induced by CCl_4_.

### 3.2. TACS Inhibited Fibrotic Protein Expression in CCl_4_-Induced Rats

**Figure 2:**
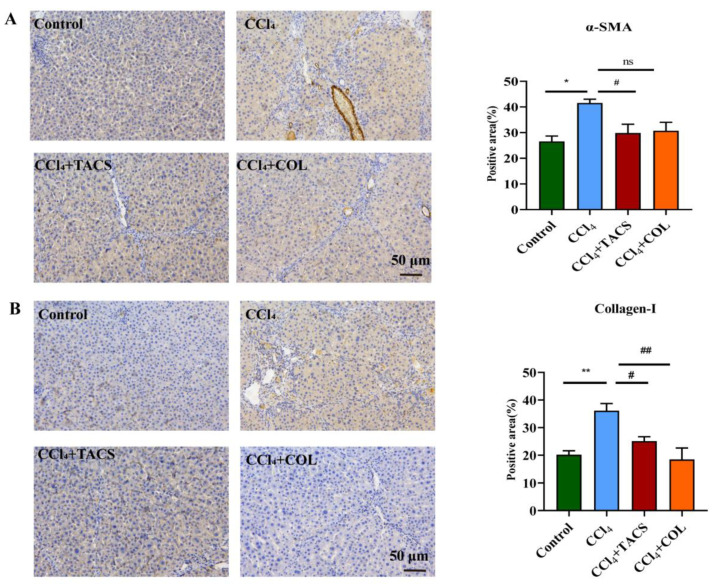
TACS inhibited α-SMA and collagen-I expression in CCl_4_-induced rats. (**A**) α-SMA immunohistochemistry representative photos and the positive area of α-SMA expression (*n* = 3). (**B**) Collagen-Ι immunohistochemistry representative images and the positive area of collagen-Ι expression (*n* = 3). Data were expressed as the mean ± SEM. * *p* < 0.05, ** *p* < 0.01 vs. control group; ^#^ *p* < 0.05, ^##^ *p* < 0.01 vs. CCl_4_-treated group, ns represents no significance.

Proteins α-SMA and collagen-Ι are the markers of activated hepatic stellate cells (HSCs). In this study, the protein levels of α-SMA and collagen-Ι expression were obviously increased compared with the control group. However, TACS treatment could markedly reverse the elevation of α-SMA and collagen-Ι in the liver (Figure 2).

### 3.3. Metabolic Profiling of Feces Metabolites Induced by Liver Fibrosis

**Figure 3:**
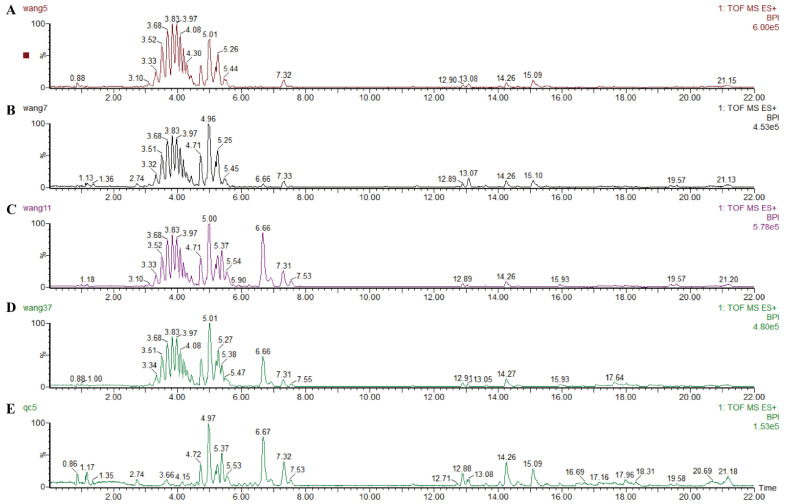
Typical BPI of feces samples in positive ESI mode. (**A**) Control group; (**B**) CCl_4_-induced liver fibrosis group; (**C**) CCl_4_ + TACS group; (**D**) CCl_4_+ COL group; (**E**) QC (quality control).

**Figure 4:**
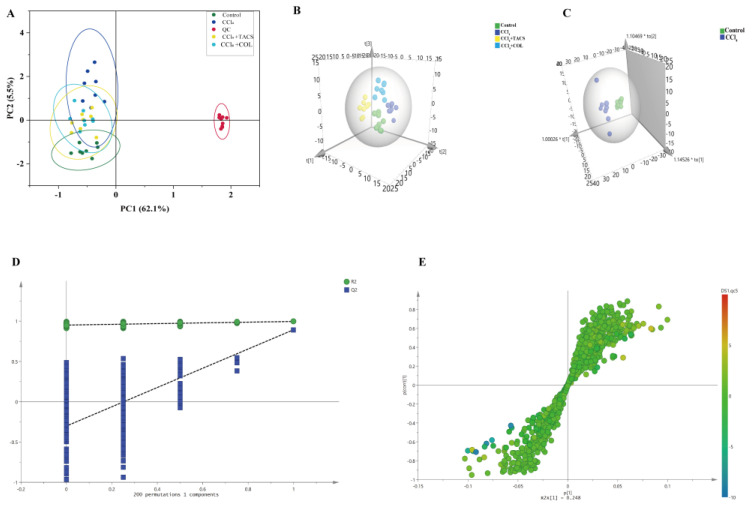
TACS intervention on CCl_4_−induced liver fibrosis rats. (**A**) PCA score plot; (**B**) PLS−DA score plot; (**C**) OPLS−DA analysis of the control and CCl_4_ groups; (**D**) Permutation test (*n* = 200 times) was used to validate the OPLS−DA model. (**E**) S−plot in control group and CCl_4_−induced liver fibrosis group detected in positive ion mode.

LC-MS was used to analyze fecal samples, and the base peak intensity (BPI) is shown in Figure 3. The feces metabolic profile was analyzed using PCA and PLS-DA. Results revealed that the metabolisms in the TACS and COL groups varied between the CCl_4_-treated group and the control group (Figure 4A,B). PC1 (62.1%) and PC2 (5.5%) together explain more than 60% of the variance in the PCA score plot. The parameters of the PCA and PLS-DA model are shown in Tables S3 and S4. These results showed that TACS treatment could regulate the level of fecal metabolites in rats with liver fibrosis. Moreover, we also observed the obvious aggregation of QC samples, indicating that the instrumental analysis was stable and feasible.

### 3.4. Identification of Potential Biomarkers for Liver Fibrosis

**Figure 5:**
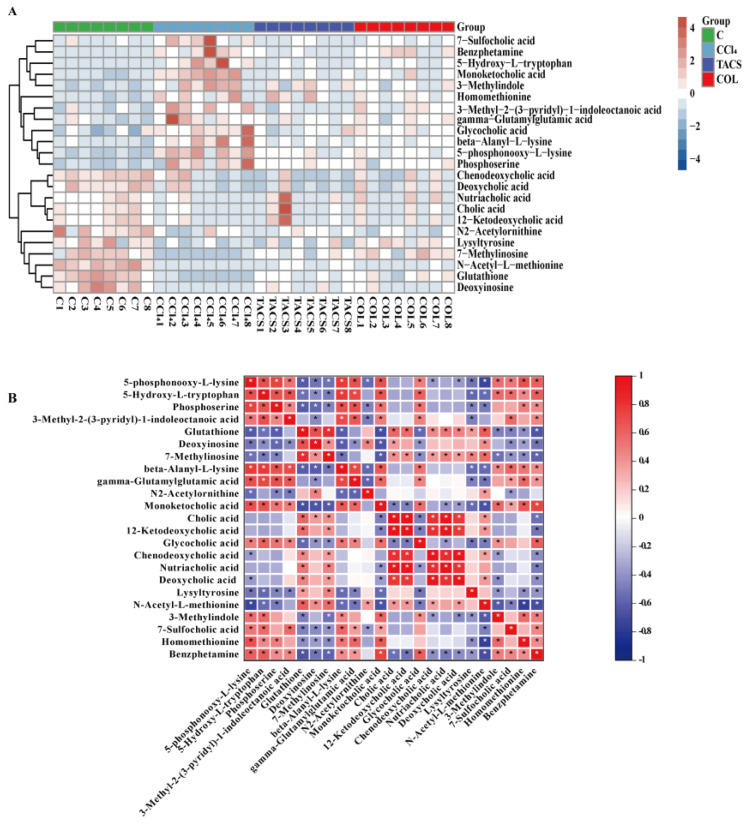
Identification of potential biomarkers for liver fibrosis. (**A**) Heatmap of the differential metabolites of liver fibrosis affected by TACS and COL. Blue: down−regulated; red: up−regulated (*n* = 8). (**B**) Spearman correlations between the differential metabolites. Red: significant positive correlations, blue: significant negative correlations. * *p* < 0.05.

In order to further investigate the potential metabolites of metabolic disorders in rats with liver fibrosis, OPLS-DA was performed to observe the separation of the CCl_4_-treated group and the control group (Figure 4C). Results indicated that endogenous metabolisms were disturbed in rats with liver fibrosis. The parameters of the OPLS-DA model are shown in Table S5. Furthermore, to prevent overfitting of the model between the control and CCl_4_-treated group, a 200-times permutation test was employed (Figure 4D). The Q^2^ values < 0.05, which indicates that the OPLS-DA model was stable and reliable. Significantly, S-plot and VIP values were applied to screen metabolites in the candidates’ feces (Figure 4E). With *p* < 0.05 and VIP > 1 as the standard, a total of twenty-three potential biomarkers were associated with liver fibrosis (Figure S4, Table 1). The semi-quantitative data of the potential biomarkers were shown in Table S6. A heat map of these 23 potential biomarkers showed that they had significant changes in the CCl_4_-treated and TACS groups (Figure 5A). To investigate the correlation between the metabolites, Spearman’s rank–order correlation analysis was performed (Figure 5B). Red and blue represent positive and negative correlations, respectively. In addition, the darker the color, the stronger the correlation between the two biomarkers.

In order to further assess the anti-hepatic fibrosis efficacy of TACS and COL, PLS-DA was applied to reveal fecal metabolic profile changes in the control, CCl_4_-treated, TACS and COL treatment groups, which revealed that TACS and COL could regulate metabolic variations induced by liver fibrosis (Figure 4B). Moreover, the OPLS-DA results indicated that the metabolic profiles of TACS and COL groups were also clearly separated from those of the CCl_4_-treated group (Figure S3A,C), and the 200-times permutation test indicated that the model was not overfitted (Figure S3B,D). Furthermore, the levels of potential differential metabolites were affected by TACS and COL intervention (Figure S4). Both TACS and COL could partly reduce the metabolic disorder in liver fibrosis rats.

### 3.5. Metabolic Pathway Analysis

**Figure 6:**
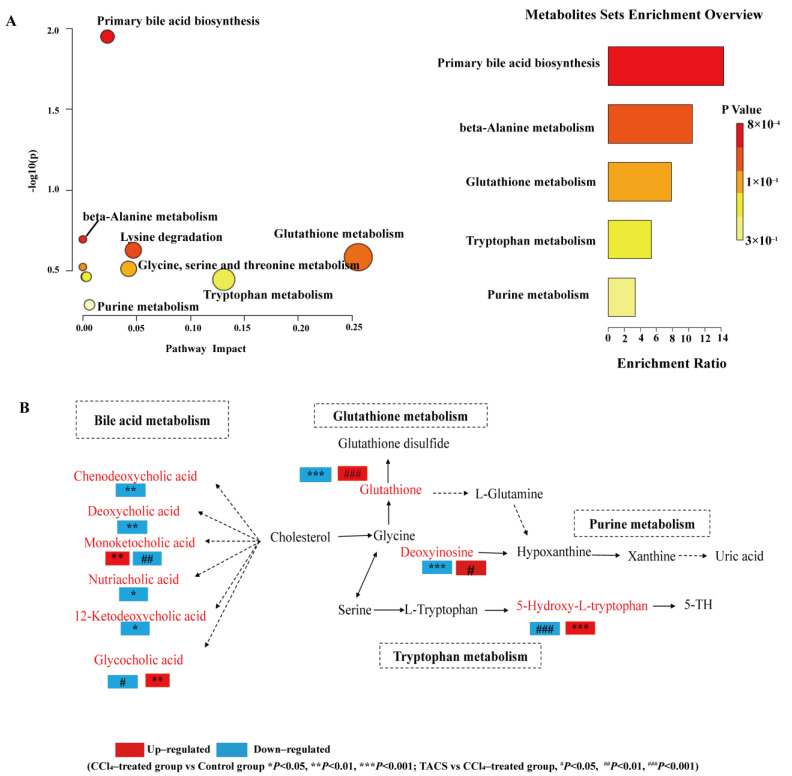
Metabolic pathway analysis (**A**) Summary of pathway analysis for potential biomarkers with MetaboAnalyst5.0. Each point represents a metabolic pathway. (**B**) Metabolic pathway networks related to the differential metabolites. Red text indicates differential metabolites. Dashed lines: indirect relationships, solid lines: direct relationships.

To investigate the metabolic pathways of TACS in liver fibrosis rats, these potential biomarkers were imported into MetaboAnalyst5.0 for further biological function analysis. Differential metabolic pathways were mapped using MetaboAnalyst5.0. As shown in Figure 6A, seven key metabolic pathways were associated with primary bile acid biosynthesis, lysine degradation, glycine, serine and threonine metabolism, glutathione metabolism, tryptophan metabolism, cysteine and methionine metabolism and purine metabolism. According to the pathway *p*-value < 0.05 or impact value > 0.1, three pathways were obviously affected in liver fibrosis rats, including glutathione metabolism, tryptophan metabolism and primary bile acid biosynthesis. The relevant parameters of all the pathways are shown in Table S7. Moreover, the corresponding metabolic pathway was mapped using KEGG, MetaboAnalyst5.0 and other online websites (Figure 6B).

### 3.6. Network Pharmacology Analysis

**Figure 7:**
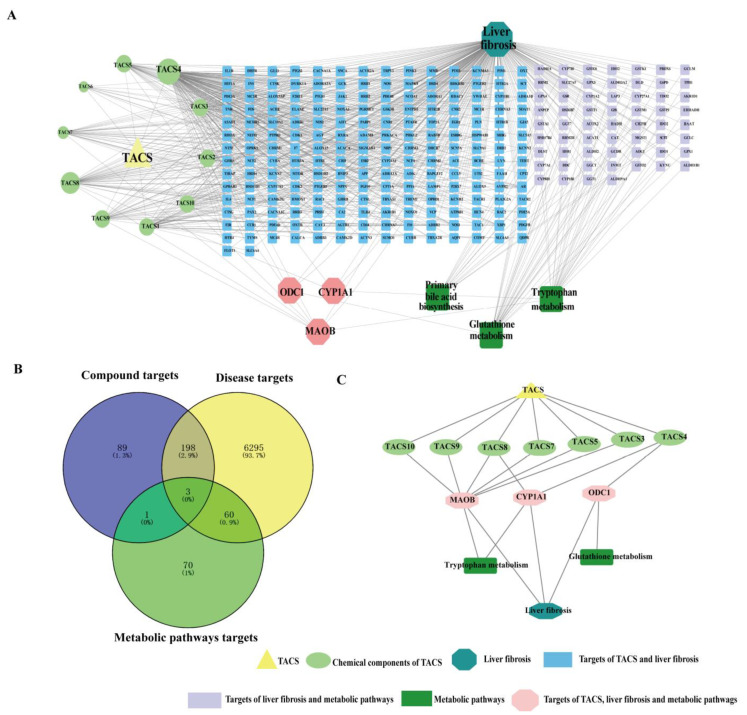
Network pharmacology prediction. (**A**)The compound–target–pathway—disease network. (**B**) The compound–target–pathway—disease Venn (**C**) Active ingredient-corresponding target–pathways–disease interaction network.

A chemical composition–target–pathways–disease network was mapped and constructed using Cytoscape3.7.2. As shown in Figure 7A, 201 potential intersection targets of TACS and disease targets were revealed based on network pharmacology (Figure 7B). In order to obtain the key targets of TACS on anti-fibrosis, ingredient–target interaction networks and PPI networks were constructed by Cytoscape3.7.2 (Figure S5). According to the criterion of being greater than the median values of the interface, tightness and degree, some core targets were identified. As shown in Table S8, Toll-Like Receptor 4 (TLR4), Interleukin 6 (IL-6) and Recombinant Prostaglandin Endoperoxide Synthase 2 (PTGS2) were considered as predicated targets for component-related targets and disease targets. Moreover, CYP1A1, ODC1 and MAOB may be potential targets related to the anti-liver fibrosis effects of dehydrocarptin, tetrahydropalmatine and palmatine, respectively (Figure 7C).

### 3.7. Molecular Docking

**Figure 8:**
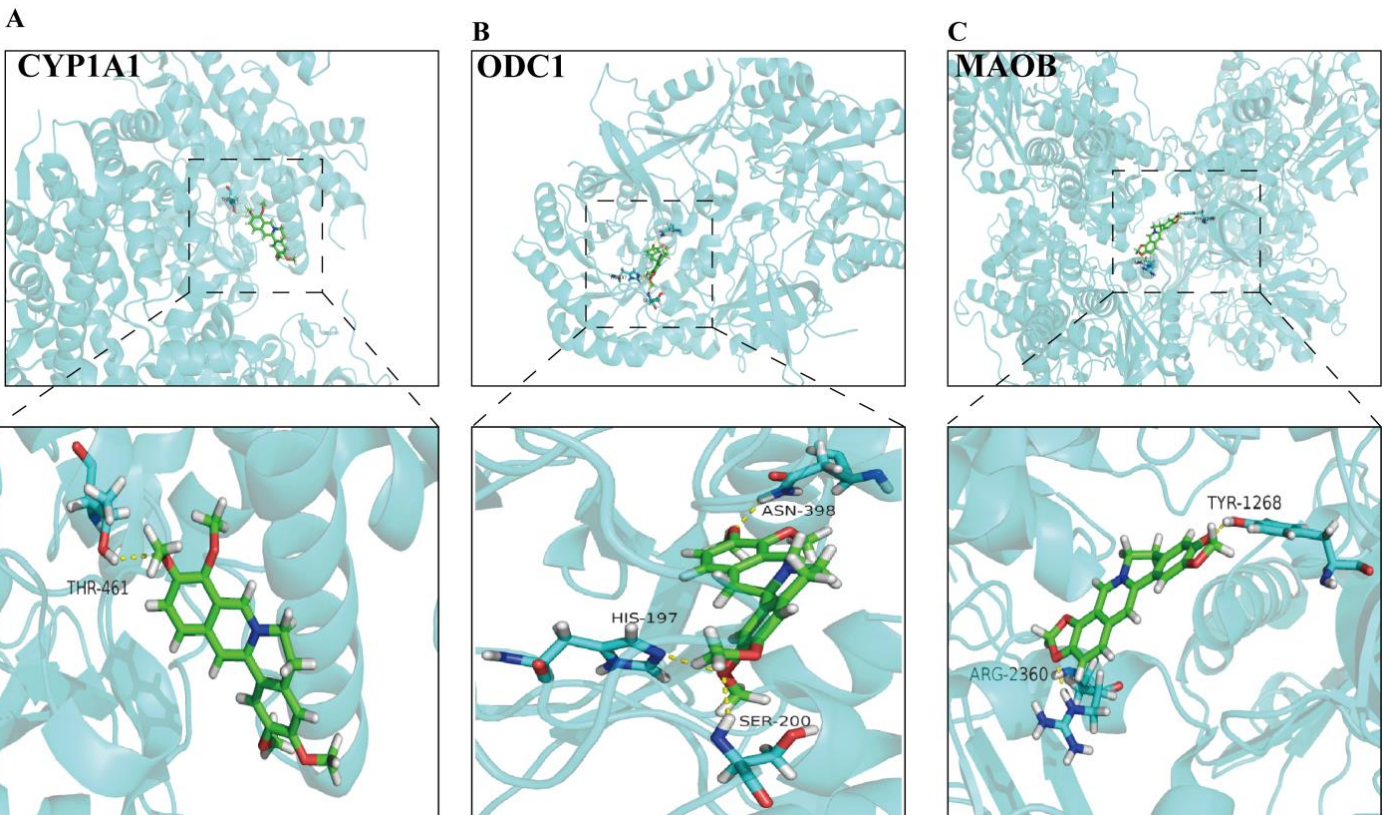
Interaction diagrams of active ingredients and key targets were scored by SYBYL-X version 2.0 and PyMoL. (**A**) Palmatine; (**B**) Tetrahydropalmatine; (**C**) Dehydrocavidine.

Molecular docking was applied to deeply explore the possibility of interaction between TACS (dehydrocavidine, tetrahydropalmatine and palmatine) and potential key targets. Our findings show that MAOB and dehydrocavidine, ODC1 and tetrahydropalmatine, CYP1A1 and palmatine have a strong affinity (Figure 8). The smaller the value of the docking score of the compound and the target, the worse the binding activity will be [21]. The number of conformations docked for each compound with free binding energy lower than −17 kcal/mol were shown in Table S9.

## 4. Discussion

Liver fibrosis is a pathological result of the wound-healing response caused by chronic liver injury [22]. The anti-hepatic fibrosis efficacy of TACS is unclear. In this experiment, we established a CCl_4_-induced liver fibrosis model to study the anti-liver fibrosis effects of TACS. Colchicine was used as the positive control drug owing to its widespread use in therapy for liver fibrosis [23]. LC-MS metabolomics combined with network pharmacology were applied to study the anti-liver fibrosis mechanisms of TACS.

AST and ALT are usually used to reflect the degree of hepatocyte injury. Liver cell degeneration occurred and the liver cell membrane was damaged under CCl_4_ stimulation; AST and ALT were released into the blood through the damaged liver cell membrane, which led to an abnormal increase in serum levels of AST and ALT [24]. In our research, the levels of ALT and AST were obviously increased, indicating the liver damage caused by CCl_4_ [25]. Our results demonstrated that TACS could improve liver function by modulating AST and ALT in liver fibrosis rats. Based on this, AST and ALT can be used as an auxiliary index to evaluate liver fibrosis. CCl_4_-induced liver fibrosis is related to lipid peroxidation. MDA and SOD are some of the vital indicators of oxidative stress. With the increase in MDA concentration, the degree of cell membrane lipid peroxidation damage was greater [26]. In addition, SOD is the main antioxidant enzyme in cells, and a reduction in its activity can lead to the accumulation of oxygen free radicals, thereby leading to cell necrosis [27]. In this study, TACS reversed abnormal changes in MDA and SOD levels induced by liver fibrosis, suggesting that TACS exerts hepatoprotective effects by inhibiting lipid peroxidation and inflammatory reactions. Masson staining plays a very important role in evaluating the success of the liver fibrosis model [27]. It is very effective for the demonstration of fibrosis owing to its ability to stain collagen fibers. Masson staining showed significant fibrosis of liver tissue after CCl_4_ modelling, suggesting successful modelling of liver fibrosis caused by CCl_4_. After TACS treatment, hepatic fibrosis was clearly alleviated, indicating that TACS can effectively reduce liver fibrosis. The activated HSCs played a significant role in the process of liver fibrosis, which is associated with the activation of α-SMA and collagen-Ι accumulation [28]. In our research, TACS treatment could decrease protein levels of α-SMA and collagen-Ι in liver fibrosis rats. These results indicate that TACS improves liver fibrosis by reducing the activation and proliferation of HSCs.

Liver fibrosis is associated with metabolic disturbances. Several metabonomic studies have shown that the urine and plasma metabolite profiles of rats with liver fibrosis changed significantly [5,29]. Our study confirmed that TACS can effectively treat liver fibrosis. Based on this, the LC-MS metabolomics technique was applied to explore the changes in metabolites in rats with liver fibrosis after TACS administration and the metabolic pathway of significant enrichment. Furthermore, we drew a comprehensive metabolic network diagram to reveal the relationship between differential metabolites and metabolic pathways (Figure 6B).

### 4.1. Bile Acid Metabolism

Bile acids are key end products of cholesterol metabolism. Primary bile acids are synthesized in the liver, secreted by hepatocytes, and converted into secondary bile acids by intestinal flora, promoting intestinal nutrient absorption and maintaining bile acid homeostasis (Figure 6B). Bile acids mainly reside in the enterohepatic circulatory system and regulate lipid metabolism and microbiota composition by recycling [30,31]. Several studies have shown that bile acid metabolism is disturbed with liver fibrosis [32] and that this disruption leads to intestinal microbiota dysregulation that destroys the intestinal barrier function, causing intestinal inflammation [33,34]. We hypothesized that this result was due to the intestinal microbiota disorder caused by liver fibrosis that may destroy the intestinal barrier function. The destruction of the intestinal barrier leads to various metabolites in the intestinal lumen such as bile acids, lipopolysaccharide (LPS), etc., into the body, thereby aggravating liver fibrosis [35]. A clinical study showed that nutriacholic acid was significantly decreased in patients with hepatocellular carcinoma and can be regarded as one of the independent risk factors for hepatocellular tumor recurrence [36]. In our study, the level of nutriacholic acid was decreased in the CCl_4_-treated group; this change is consistent with other reports. Glycholic acid (GCA) is a main diagnostic indicator of liver disease [37]. Kang et al. found that the level of GCA in patients with liver fibrosis was significantly increased and had the strongest correlation with fibrosis [38]. GCA is an endogenous ligand of FXR in the gut. Xu et al. found that L-Theanine can increase the level of intestinal GCA by regulating intestinal bacterial activity associated with bile–salt hydrolase (BSH), which can inhibit the ileal FXR-FGF15 and regulate the classical BA metabolic pathway, thus maintaining the homeostasis of intestinal and hepatic circulation [39]. In this study, the level of GCA was significantly increased in the CCl_4_-treated group. After TACS treatment, the level of GCA was restored. Previous research has also shown that TACS could regulate the disturbance of bile acid metabolism caused by antibiotics [19]. Therefore, we hypothesized that TACS may exert an anti-fibrosis effect by regulating bile acid synthesis and metabolism.

### 4.2. Glutathione Metabolism

Glutathione (GSH) is an important non-enzymatic antioxidant which plays a role in protecting the intestinal tract [40] and the liver from oxidative damage [41]. Recent research has shown that liver fibrosis can lead to the destruction of intestinal mucosa [42] and the weakening of intestinal glutathione-dependent antioxidant defense, thereby causing decreased levels of glutathione [43]. It has also been reported that GSH is the main intracellular detoxifying molecule, which works against toxins generated in the lumen and protects the intestinal barrier [44]. In this work, the level of glutathione was decreased in the CCl_4_-treated group, indicating a disordered glutathione metabolism. This may be the result of intestinal inflammation caused by liver fibrosis. In addition, increased intestinal permeability leads to liver toxic substances (such as LPS) entering into the liver [45] and promotes intestinal bacterial translocation [46], thereby activating the hepatic inflammatory pathway and aggravating hepatic fibrosis. Nevertheless, the downregulation of glutathione was reversed by TACS intervention, indicating that TACS could ameliorate the disturbed glutathione metabolism.

### 4.3. Tryptophan Metabolism

The compound 5-hydroxy-L-tryptophan is an intermediate in the synthesis of serotonin from tryptophan (Figure 6B). Tryptophan is converted to 5-hydroxy-L-tryptophan under the catalysis of tryptophan hydroxylase 1 (TPH1). Previous research has shown that TPH1 is associated with gut microbes, especially gut probiotics [47]. Based on this, we speculate that intestinal flora disorders caused by liver fibrosis may inhibit the activity of TPH1, thus hindering the normal metabolism of tryptophan and leading to an abnormal increase in 5-hydroxy-L-tryptophan. Wrzosek et al. found that the level of tryptophan was significantly decreased in patients with severe alcoholic hepatitis compared with healthy people, and pectin can reduce liver damage in mice by increasing tryptophan levels and activating the AHR pathway [48]. Therefore, we speculate that TACS may reduce liver fibrosis by indirectly increasing the level of tryptophan through regulating TPH1 activity and restoring the level of 5-hydroxy-L-tryptophan.

### 4.4. Purine Metabolism

Deoxyinosine produces hypoxanthine through the action of purine nucleoside phosphorylase, which then reacts with xanthine oxidase to form xanthine and uric acid (Figure 6B). Uric acid is an antioxidant molecule that plays an indispensable role in the gut and liver [49]. High levels of uric acid lead to intestinal inflammation and liver damage by activating the inflammatory and apoptotic pathways [50]. In this study, deoxyinosine was significantly decreased in the CCl_4_-treated group, indicating a disorder in the purine metabolism. We speculated that this may be caused by intestinal oxidative stress after CCl_4_ administration, and that this reaction promotes the decomposition of deoxyinosin towards uric acid [51]. The fecal level of deoxyinosine in TACS group increased, indicating that TACS may significantly up-regulate the level of deoxyinosine by alleviating intestinal inflammation, thus reducing liver fibrosis. Therefore, those results indicated that TACS could regulate the disorder of multiple metabolic pathways caused by liver fibrosis.

In order to further study the complex mechanism of TACS on anti-fibrosis effects, we integrated the network pharmacology and metabolomics data to obtain a new perspective. We used UPLC-Q-TOF/MS to characterize TACS and identified 10 main compounds. Numerous studies have demonstrated that Berberrubine [52], Tetrahydropalmatine [53], Jatrorrhizine [54], Coptisine [55], Dehydrocavidine [10], Palmatine [56], Berberine [57] and Chelerythrine [5] are associated with liver damage. CYP1A1, ODC1 and MAOB were considered as targets of TACS by metabolomics and network pharmacology. Meanwhile, dehydrocavidine, tetrahydropalmatine and palmatine are also considered as potential active compounds of TACS to therapeutic hepatic fibrosis. Dehydrocavidine can attenuate CCl_4_-induced liver fibrosis by reducing oxidative stress and modulating fibrosis-related genes [10]. Xuan et al. found that tetrahydropalmatine can protect mice from liver fibrosis through inhibiting endoplasmic reticulum stress in hepatic stellate cells [53]. In addition, studies have shown that palmatine can reduce liver injury through suppressing hepatocyte apoptosis by the autophagy pathway [56,58]. CYP1A1, as a microsomal enzyme, participates in fatty acid oxidation. Previous studies have shown that CYP1A1 plays a vital role involved in the oxidative stress response. Meanwhile, CYP1A1 attenuates lipid peroxidation and liver damage by affecting the levels of MDA, SOD and ROS [59,60]. ODC1 is a metabolic enzyme that plays a role in polyamine biosynthesis. Previous research has shown that ODC1 promotes hepatocellular carcinoma (HCC) proliferation by affecting the AKT/GSK3β/β-catenin pathway [61]. It has been reported that the target of ODC1 may be an effective therapeutic method for HCC [62]. MAOB plays a vital role in hepatic fibrosis. Many researchers have reported that MAOB was involved in the formation of collagen fibers [63,64]. MAOB is considered an ideal marker for the diagnosis of hepatic fibrosis, and the level of MAOB was obviously increased in the sera of patients with early liver fibrosis [65]. Therefore, our results demonstrated that network pharmacology combined with metabolomics may provide a new perspective to explore hepatic fibrosis mechanisms based on multiple components and multiple targets of TACS.

However, our research still has many limitations. Based on our findings, firstly, it is still unclear whether the changes in these metabolites are the cause or the effect. In addition, network pharmacology and molecular docking provide only preliminary exploration of potential active ingredients and targets, and we need to further study molecular mechanisms by molecular and biological pathways.

## 5. Conclusions

In this study, metabolomics and network pharmacology were used to explore the anti-liver fibrosis mechanisms of TACS. Compared with the control group, 23 metabolites with obvious changes in the CCl_4_-treated group were identified. Additionally, the metabolic pathways related to liver fibrosis mainly included primary bile acid biosynthesis, glutathione metabolism, tryptophan metabolism and purine metabolism. A total of 201 potential intersection targets of TACS and liver fibrosis were revealed based on network pharmacology. Furthermore, CYP1A1, ODC1 and MAOB comprise the intersection of TACS targets, metabolic pathway targets and disease-related targets, and dehydrocavidine, tetrahydropalmatine and palmatine are considered as potential active compounds for CCl_4_-induced liver fibrosis therapy. Our research provides data support in order to further explore the mechanism of TACS in treating liver fibrosis in the future, and it also lays a foundation for the pharmacological study of *C. saxicola* Bunting.

## Figures and Tables

**Table 1:** The potential biomarkers of feces in liver fibrosis rats (*n* = 8).

N0	RT	m/z	VIP	PPM	MS/MS	Metabolites	Formula	Adduct Ion	Pathway	CCl_4_/C	TACS/CCl_4_	COL/CCl_4_
1	0.82	207.0543	1.49829	1	128.0755, 99.0988	5-phosphonooxy-L-lysine	C_6_H_15_N_2_O_6_P	[M+H-2H_2_O]^+^	Lysine degradation	↑ **	↓ #	↓ #
2	0.83	185.0733	1.68887	7	173.0751, 158.0673, 74.0245	5-Hydroxy-L-tryptophan	C_11_H_12_N_2_O_3_	[M+H-2H_2_O]^+^	Tryptophan metabolism	↑ **	↓ ##	↓ #
3	0.86	149.9975	1.11901	9	74.0280, 125.0015	Phosphoserine	C_3_H_8_NO_6_P	[M+H-2H_2_O]^+^	Cysteine and methionine metabolism, Glycine, serine and threonine metabolism	↑ *	↓ #	−
4	1	176.1076	1.3219	3	95.0980, 97.1038	3-Methyl-2-(3-pyridyl)-1-indoleoctanoic acid	C_22_H_26_N_2_	[M+2H]^+^	−	↑ **	↓ ###	↓
5	1	325.1163	1.5421	4	290.0865, 274.1086, 264.1042, 233.0599, 216.0840, 205.0615, 86.0639	Glutathione	C_10_H_17_N_3_O_6_S	[M+NH_4_]^+^	Glutathione metabolism	↓ ***	↑ ##	↑ ##
6	1.01	522.2034	1.80521	4	253.0923, 226.0811, 191.0567, 137.0474, 110.0374	Deoxyinosine	C_10_H_12_N_4_O_4_	[2M+NH_4_]^+^	Purine metabolism	↓ ***	↑ #	−
7	1.02	300.1328	1.5686	7	96.0595, 108.0514, 123.0606, 153.0701	7-Methylinosine	C_11_H_15_N_4_O_5_	[M+NH^4^]^+^	−	↑ ***	↓ ###	↓ ###
8	1.13	118.0914	3.1785	4	58.0672, 129.1021, 155.1189	beta-Alanyl-L-lysine	C_9_H_19_N_3_O_3_	[M+H+NH_4_]^+^	beta-Alanine metabolism	↑ ***	↓ ##	↓ #
9	1.17	139.0553	1.10885	1	102.0579, 86.0640, 84.0492	gamma-Glutamylglutamic acid	C_10_H_16_N_2_O_7_	[M+2H]^+^	−	↓ **	↑ #	−
10	1.17	174.1215	1.0145	9	158.0818, 140.0777, 129.1059, 116.0757, 112.0769, 85.0762, 60.0808	N2-Acetylornithine	C_7_H_14_N_2_O_3_	[M+NH_4_-H_2_O]^+^	−	↓ **	↑	−
11	3.51	327.205	1.16328	7	310.1781, 182.0842, 136.0775, 129.1010, 95.0645, 84.0857	Lysyltyrosine	C_15_H_23_N_3_O_4_	[M+NH_4_]^+^	−	↓ **	↑ #	↑ ##
12	4.23	174.0599	1.60695	6	156.0491, 146.0647, 132.0499, 126.0599, 104.0569	N-Acetyl-L-methionine	C_7_H_13_NO_3_S	[M+H-H_2_O]^+^	−	↓ **	−	−
13	4.35	245.143	1.64268	3	106.0655, 79.0510, 132.0871	3-Methylindole	C_9_H_9_N	[2M+H-H_2_O]^+^	−	↑ *	−	↓ #
14	4.36	506.2784	1.11455	0	489.2525, 471.2425, 367.1596, 85.0687, 55.0588	7-Sulfocholic acid	C_24_H_40_O_8_S	[M+NH_4_]^+^	−	↑ *	↓ #	↓
15	4.5	164.0756	1.55243	10	70.0697, 160.0727	Homomethionine	C_6_H_13_NO_2_S	[M+H]^+^	−	↑ **	−	−
16	4.57	303.1839	1.888	2	122.0970, 120.0836, 93.0645	Benzphetamine	C_17_H_21_N	[M+ACN+Na]^+^	−	↑ **	↓ ##	↓
17	12.26	423.2771	1.6011	7	421.2584, 405.2632, 345.2471, 127.0787	Monoketocholic acid	C_24_H_38_O_6_	[M+H]^+^	Bile Acid Metabolism	↑ **	↓ ##	↓ #
18	14.06	391.2866	1.79775	6	391.2873, 373.2771, 365.2665, 289.2195	Cholic acid	C_24_H_40_O_5_	[M+NH_4_-H_2_O]^+^	Bile Acid Metabolism	↓ *	↑	−
19	14.06	413.2683	1.24289	5	391.2873, 373.2771, 355.2648, 289.2195, 271.2043	12-Ketodeoxycholic acid	C_24_H_38_O_4_	[M+Na]^+^	Bile Acid Metabolism	↓ *	−	−
20	14.63	488.3015	1.23856	7	407.2729, 365.2633, 355.2450, 85.0699	Glycocholic acid	C_24_H_40_O_6_	[M+ACN+Na]^+^	Bile Acid Metabolism	↑ **	↓ #	↓ #
21	15.1	410.3289	2.75643	6	375.2928, 347.2920, 315.2688, 73.0295, 299.2342	Chenodeoxycholic acid	C_24_H_40_O_4_	[M+NH_4_]^+^	Bile Acid Metabolism	↓ *	↓	−
22	15.19	408.3129	1.60232	5	373.2743, 355.2679, 277.2149, 257.1904, 175.1107	Nutriacholic acid	C_24_H_38_O_4_	[M+NH_4_]^+^	Bile Acid Metabolism	↓ *	↑	−
23	15.53	410.3285	1.97283	5	349.2756, 301.2562, 271.2059, 243.1787, 165.1282, 147.1184, 85.0655	Deoxycholic acid	C_24_H_40_O_4_	[M+NH_4_]^+^	Bile Acid Metabolism	↓ *	−	−

* p < 0.05, ** *p* < 0.01, *** *p* < 0.001 vs. group; ^#^ *p* < 0.05, ^##^ *p* < 0.01, ^###^ *p* < 0.001 vs. CCl_4_−induced. ↑ represents up-regulated metabolite levels, ↓ represents down-regulated metabolite levels, − represents no change in metabolite levels.

## Data Availability

All data are contained in the article and Supplementary Materials.

## Supplementary Materials

The following supporting information can be downloaded at: https://www.mdpi.com/article/10.3390/metabo13010009/s1, Figure S1. BPI chromatograms of TACS (A), mixed standard (B). Jatrorrhizine (C), Coptisine (D), Dehydrocavidine (E), Palmatine (F), Berberine (G), Chelerythrine (H). Figure S2. Chemical structures of ten alkaloid components of TACS. Figure S3. (A,C) OPLS-DA score plot. (A) CCl_4_-treated group vs. TACS group; (C) CCl_4_-treated group vs. COL group; (B,D) Permutation test (*n* = 200 times) was used to validate the OPLS-DA model. (B) CCl_4_-treated group vs. TACS group; (D) CCl_4_-treated group vs. COL group. Figure S4. Levels of differential metabolites in feces after intervening with TACS and colchicine. Figure S5. The PPI network of TACS treatment on liver fibrosis. Node color reflects its degree. Table S1. Characterization and identification of compounds in TACS (positive ion mode). Table S2. The reproducibility of UPLC-Q-TOF/MS method validation under the positive ion mode using QC samples. Table S3. The parameters for evaluating the model quality of PCA in positive ion modes. Table S4. The parameters for evaluating the model quality of PLS-DA in positive ion modes. Table S5. The parameters for evaluating the model quality of OPLS-DA in positive ion modes. Table S6. Levels of differential metabolites in feces (*n* = 8). Table S7. The relevant detailed parameters in the pathway enrichment. Table S8. The core targets of TACS treatment on liver fibrosis (Top 10). Table S9. The relevant information of molecular docking.
