# Supplementary material for: Investigation of the Therapeutic Effect of Total Alkaloids of Corydalis saxicola Bunting on CCl4-Induced Liver Fibrosis in Rats by LC/MS-Based Metabolomics Analysis and Network Pharmacology
Source: Metabolites. 2022 Dec 21;13(1):9. doi: 10.3390/metabo13010009 (PMC9866371; doi:10.3390/metabo13010009)
Supplement: Supplementary file 1 [file metabolites-13-00009-s001.zip › metabolites-2054450-supplementary.pdf]

Supplementary material

Investigation of the Therapeutic Effect of Total Alkaloids of *Corydalis saxicola* Bunting on CCl_4-induced Liver Fibrosis in Rats by LC/MS-Based Metabolomics Analysis and Network Pharmacology

1.Materials and methods

1.1 Chemicals

The reference standards of Coptisine, Berberine, Palmatine, Chelerythrine, Epiberberine and Jatrorhizine were purchased from Chengdu Must Bio-Technology Co., Ltd (Chengdu, China), and the batch number were MUST-14051612, MUST-16111115, MUST-17022604, MUST-14062313, MUST-17072011 and MSUT-17041801 respectively. Dehydrocavidine was purchased from National Institute for the Control of Pharmaceutical and Biological Products, and the batch number was 11667-200401.

1.2 The extraction process of TACS

Firstly, 100 g of CS herbs was weighed into a 2 L round-bottomed flask, and 10 times the amount of 75% ethanol (1:10 *w/v*) was added for reflux extraction for 2 h and repeated extraction for 3 times, combined all the filtrate, follow by the filtrate was concentrated to 1:1 (*w/v*) by the rotary evaporator. The next step was to add 4 times the amount of 1%

hydrochloric acid (*w/v*) with reflux extraction for 1 hour, then the solution was adjusted to PH 7.0 with 40%NaOH, and the solution was concentrated to the relative specific gravity of 1.06-1.08, then the crude extract was extracted after being stored at 4 ℃for 48 h. Secondly, reflux extraction with 16 times the amount of 1% hydrochloric acid (*w/v*) was added to the crude extract for 1 h, then, the filtrate was adjusted to PH 7.0, follow by the filtrate was concentrated to the relative specific gravity of 1.06-1.08, and stored at 4 ℃ for 48 h. Finally, TACS was obtained by filtration. The compounds of TACS were identified by UPLC-Q-TOF/MS (Figure S1).

1.3 Sample preparation:

The TACS solution was dissolved by methanol to concentration of 0.5 mg/ml. The mixed standard solutions including six compounds that is Coptisine, Jatrorhizine, Dehydrocavidine, Palmatine, Berberine and Chelerythrine. The mixed standard solutions concentration of 0.05 mg/ml.

1.4 Identification of components in TACS

The chemical structures were identified by analyzing of their retention behaviors, accurate molecular weight and MSE fragment information, and confirmed by Human Metabolome Database (http://www.hmdb.ca) (accessed on 10 August 2022), the standard product database established by our group and consulting relevant literatures.

2 Results

The identification result of TACS was shown in Figure S2 and Table S1. 10 components in TACS were identified, including Cheilanthifoline, Berberrubine, Epiberberine, Tetrahydropalmatine, Jatrorrhizine, Coptisine, Dehydrocavidine, Palmatine, Berberine, Chelerythrine, which were identified by online database, standards and literature.

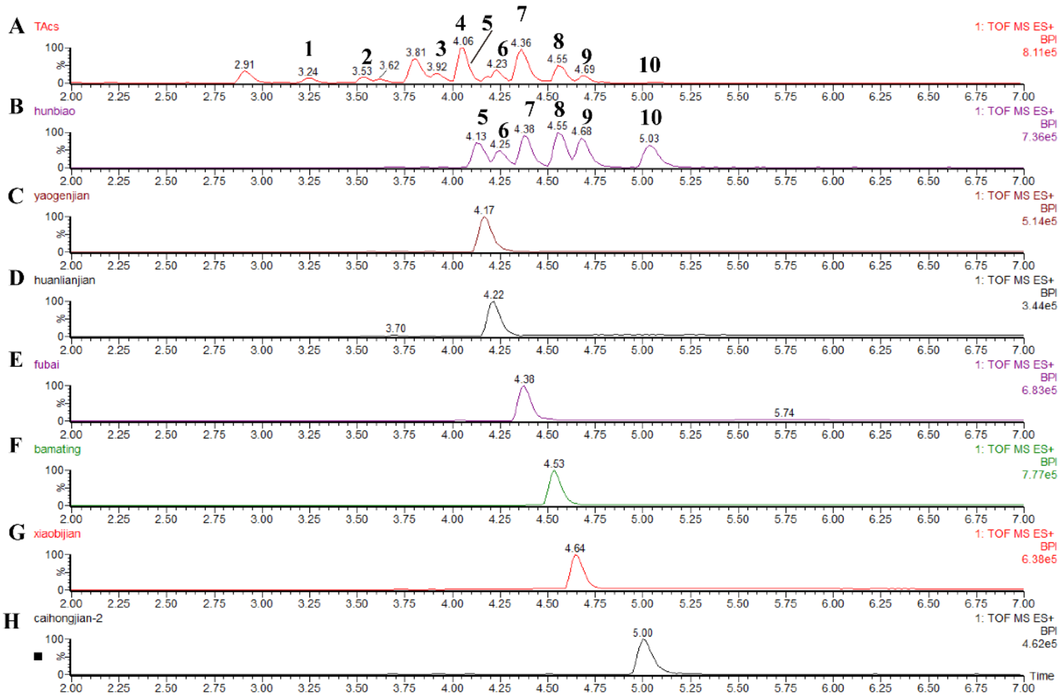

Figure S1. BPI chromatograms of TACS (A), mixed standard (B). Jatrorrhizine (C), Coptisine (D), Dehydrocavidine (E), Palmatine (F), Berberine (G), Chelerythrine (H). (1: Cheilanthifoline, 2: Berberrubine, 3: Epiberberine, 4: Tetrahydropalmatine, 5: Jatrorrhizine, 6: Coptisine, 7: Dehydrocavidine, 8: Palmatine, 9: Berberine, 10: Chelerythrine)

Table S1 Characterization and identification of compounds in TACS (positive ion mode)

NO	R_t/min	Compounds	Contents(g/g)	Formula	Adduction	Calculated mass（m/z)	Experimental mass (MS)	Error (ppm)	Fragment ion (MS/MS) m/z
TACS1	3.24	Cheilanthifoline	0.0007	$C_{19}H_{20}NO_4^+$	$[M]^+$	326.1394	326.1389	1.53	326.1389,178.0870,176.0708,163.0635,151.0759,149.0603
TACS2	3.53	Berberrubine	0.0025	$C_{19}H_{16}NO_4^+$	$[M]^+$	322.1082	322.1079	0.93	320.0939,307.0844,279.0896,251.0949
TACS3	3.92	Epiberberine	0.0174	$C_{20}H_{18}NO_4^+$	$[M]^+$	336.1241	336.1229	3.57	292.0963,278.0807
TACS4	4.06	Tetrahydropalmatine	0.0480	$C_{21}H_{26}NO_4^+$	$[M]^+$	356.1859	356.1861	-0.56	192.1026,177.0790
TACS5	4.17	Jatrorrhizine	0.0017	$C_{20}H_{20}NO_4^+$	$[M]^+$	338.1387	338.1381	1.77	322.1067,294.1136
TACS6	4.23	Coptisine	0.0050	$C_{19}H_{14}NO_4^+$	$[M]^+$	320.0918	320.0909	2.81	290.0827,262.0744,
TACS7	4.36	Dehydrocavidine	0.0327	$C_{21}H_{20}NO_4^+$	$[M]^+$	350.1387	350.1387	0.00	334.1075,306.1122,276.1010
TACS8	4.51	Palmatine	0.0254	$C_{21}H_{22}NO_4^+$	$[M]^+$	352.1544	352.1551	-1.99	336.1122,322.1077,294.1122,292.0954,278.0872
TACS9	4.69	Berberine	0.0088	$C_{20}H_{18}NO_4^+$	$[M]^+$	336.1231	336.1227	1.19	320.0917,318.0732,306.0761,290.0821,278.0820
TACS10	5.05	Chelerythrine	0.0001	$C_{21}H_{18}NO_4^+$	$[M]^+$	348.1231	348.1208	6.61	332.0930,318.0741,290.0824

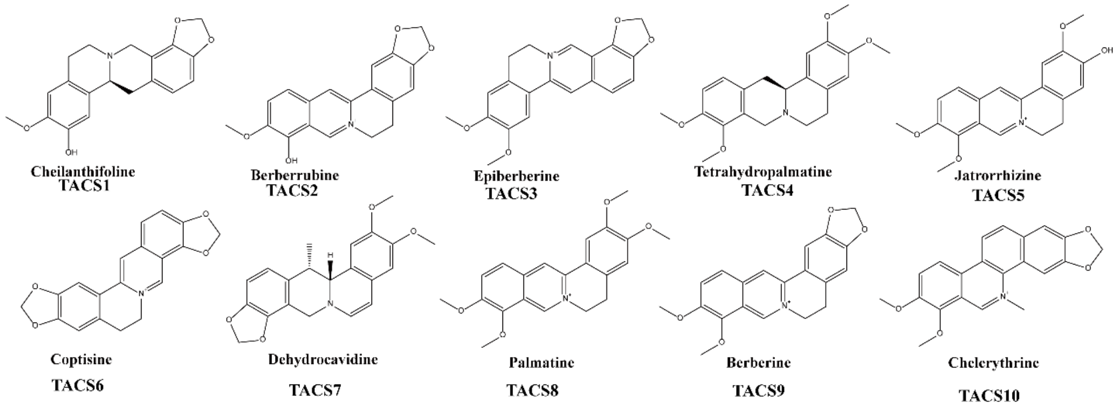

Figure S2. Chemical structures of ten alkaloid components of TACS.

The MS/MS spectra and the fragmentation process of the compounds of TACS are shown below as follows:

1.Cheilanthifoline

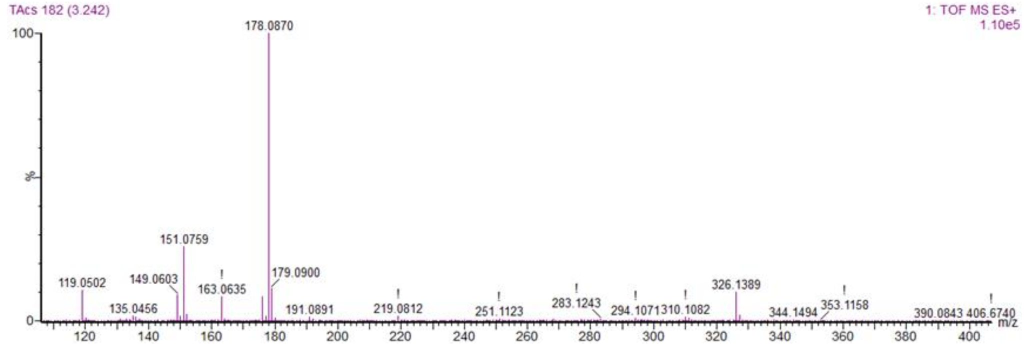

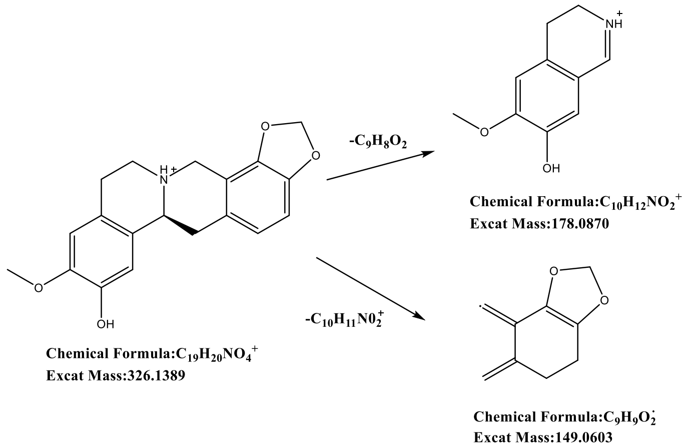

2. Berberrubine

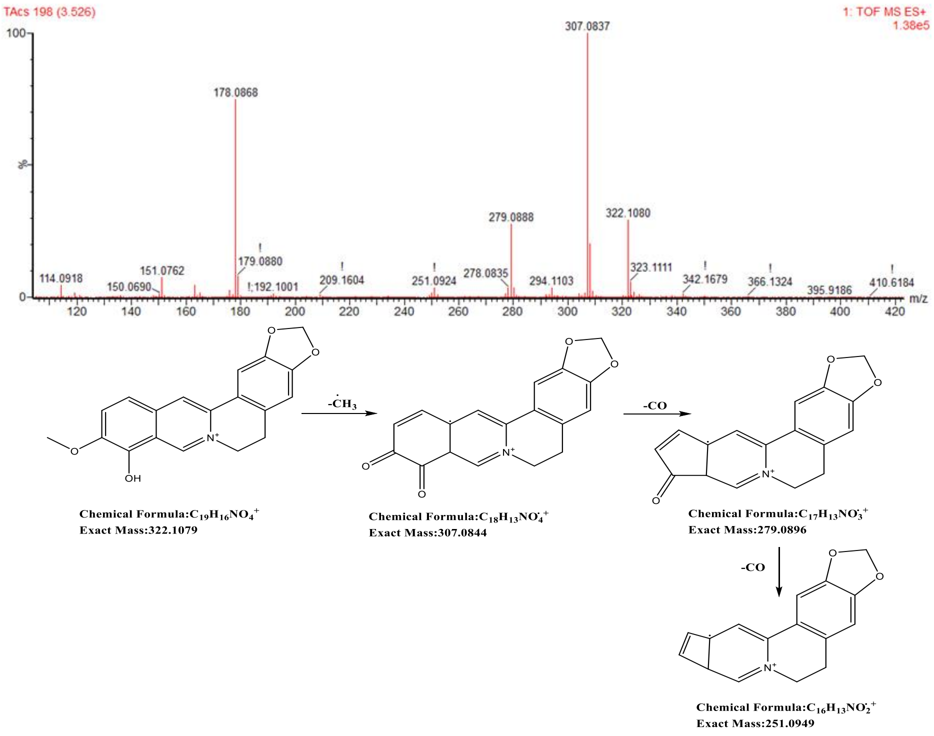

3.Epiberberine

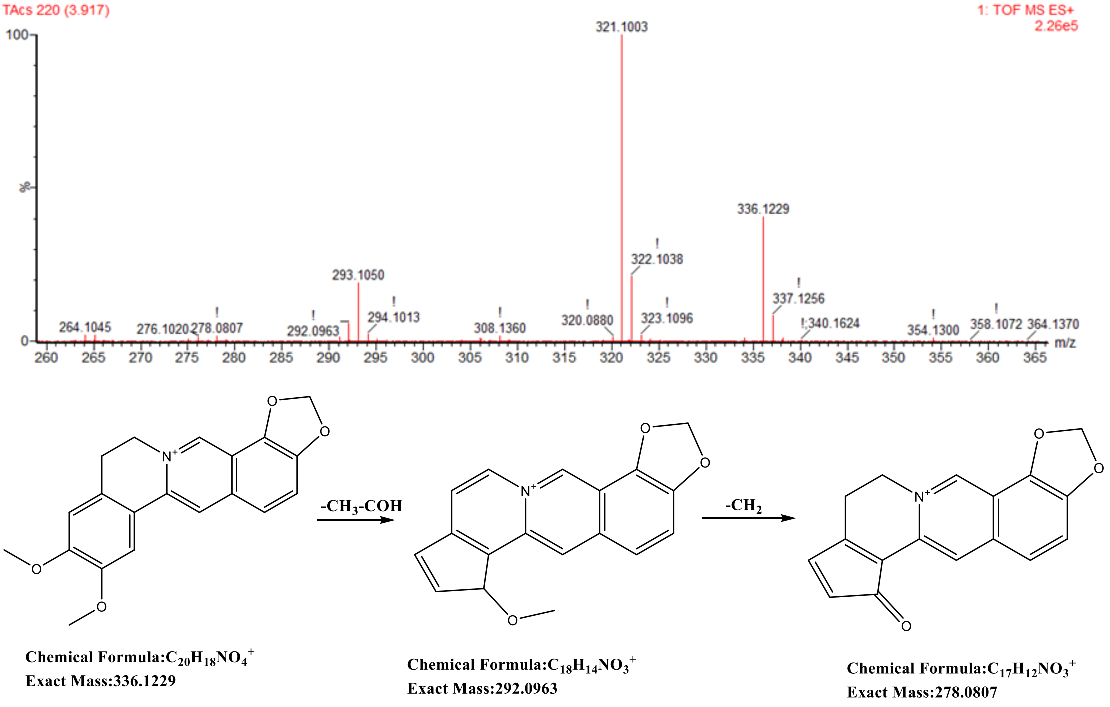

4. Tetrahydropalmatine

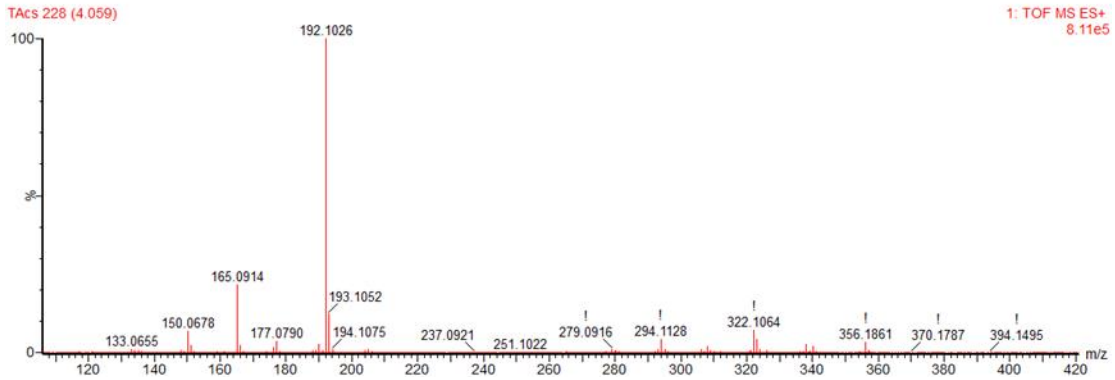

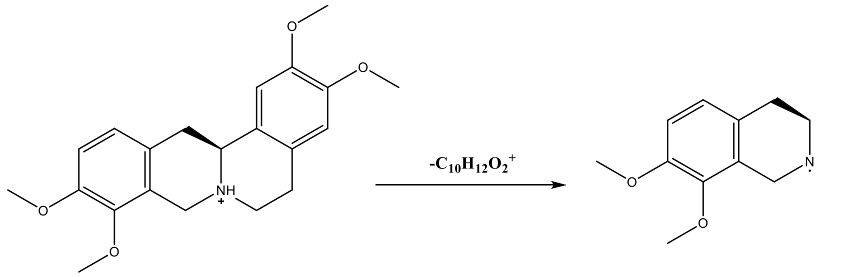

Chemical Formula: $C_{21}H_{26}NO_4^+$
Exact Mass:356.1861

Chemical Formula: $C_{11}H_{14}NO_2^{\cdot}$
Exact Mass:192.1026

5. Jatrorrhizine

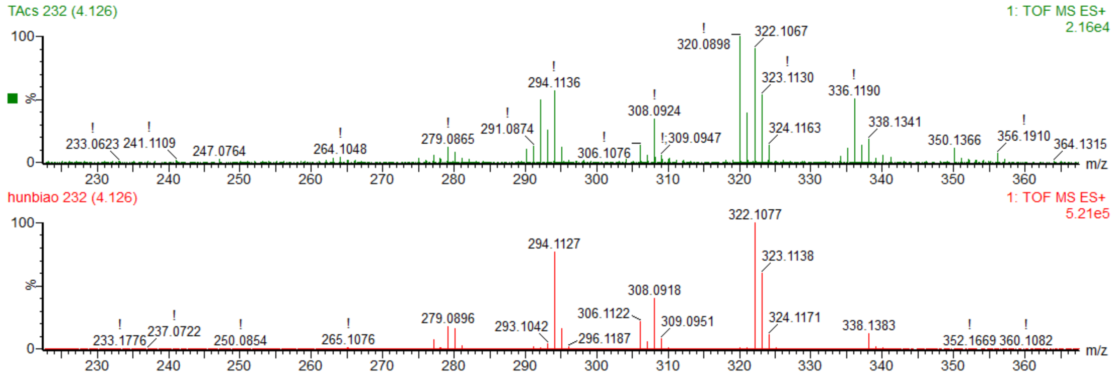

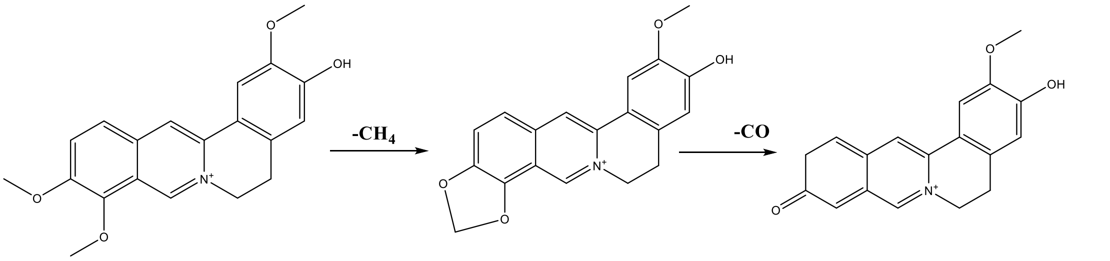

Chemical Formula: $C_{20}H_{20}NO_4^+$
Exact Mass:338.1387

Chemical Formula: $C_{19}H_{16}NO_4^+$
Exact Mass:322.1074

Chemical Formula: $C_{18}H_{20}NO_3^+$
Exact Mass:294.1125

6. Coptisine

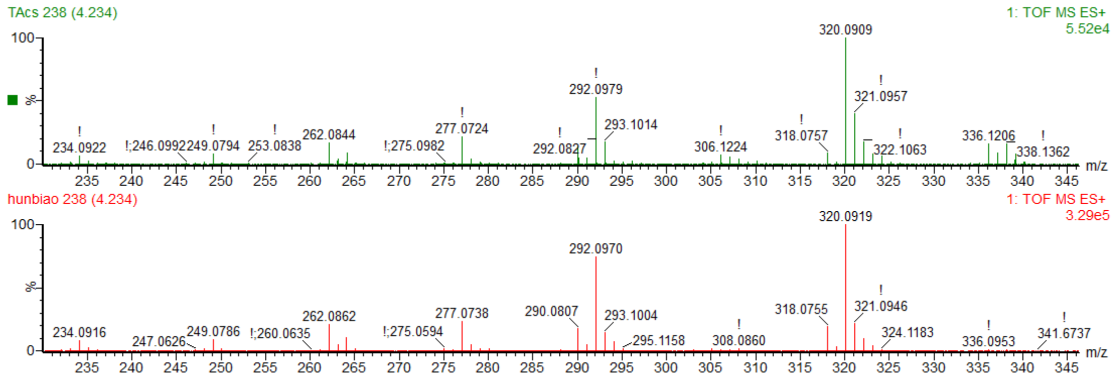

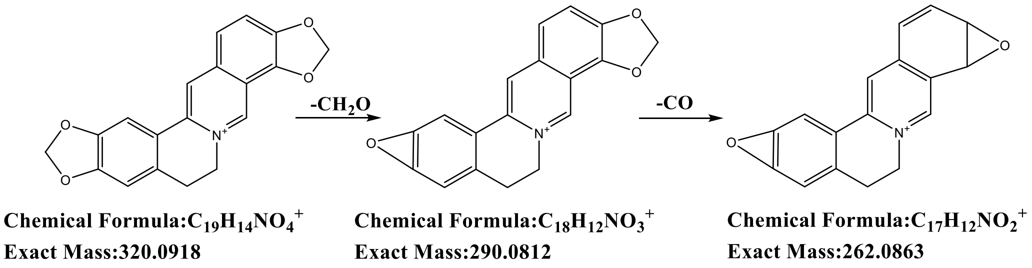

7. Dehydrocavidine

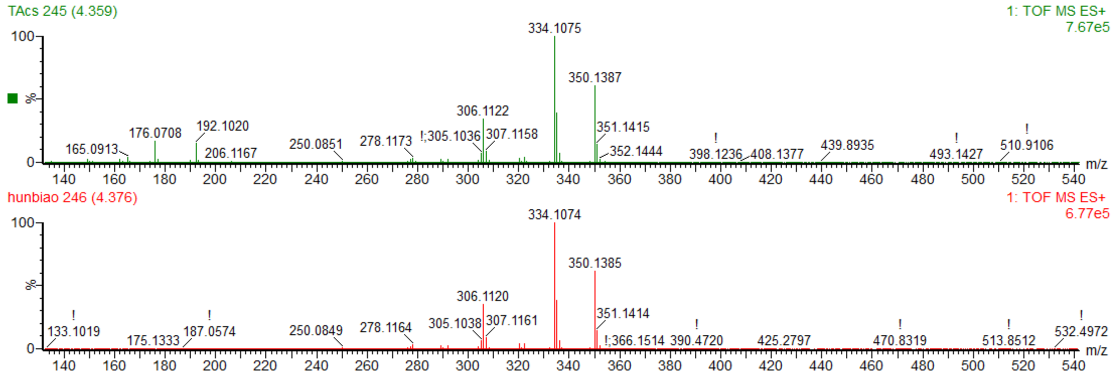

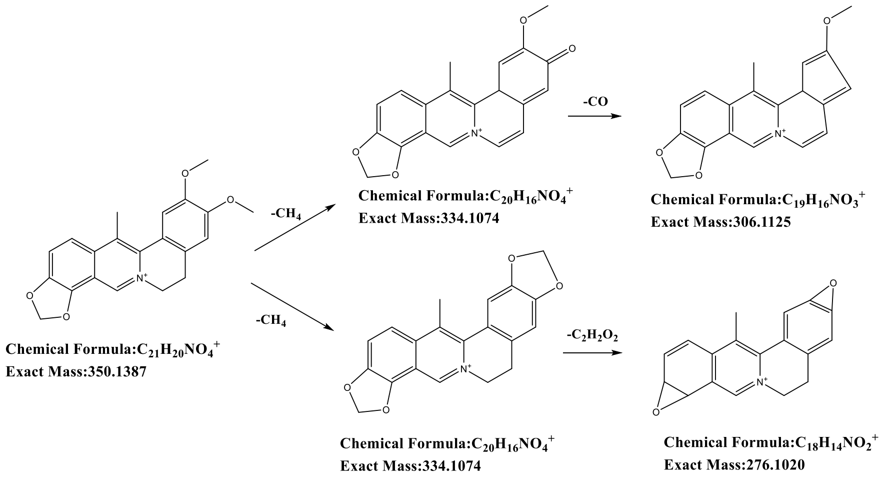

8. Palmatine

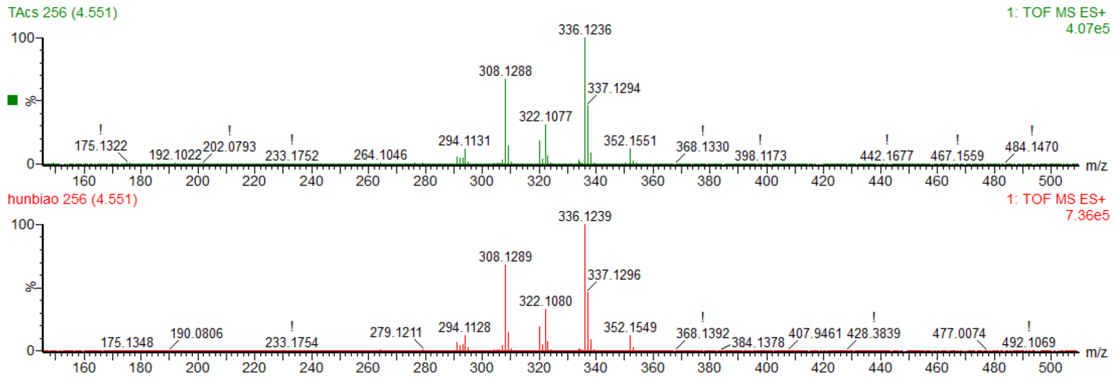

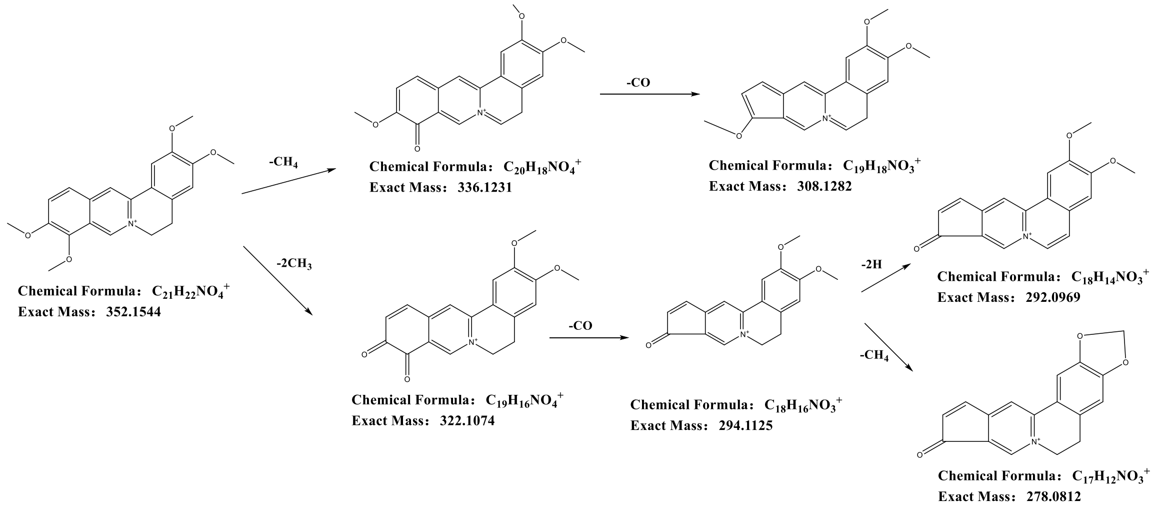

9. Berberine

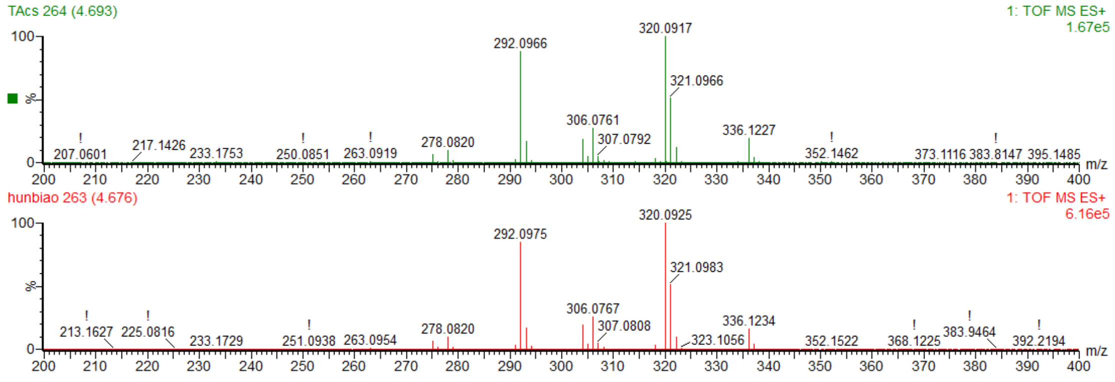

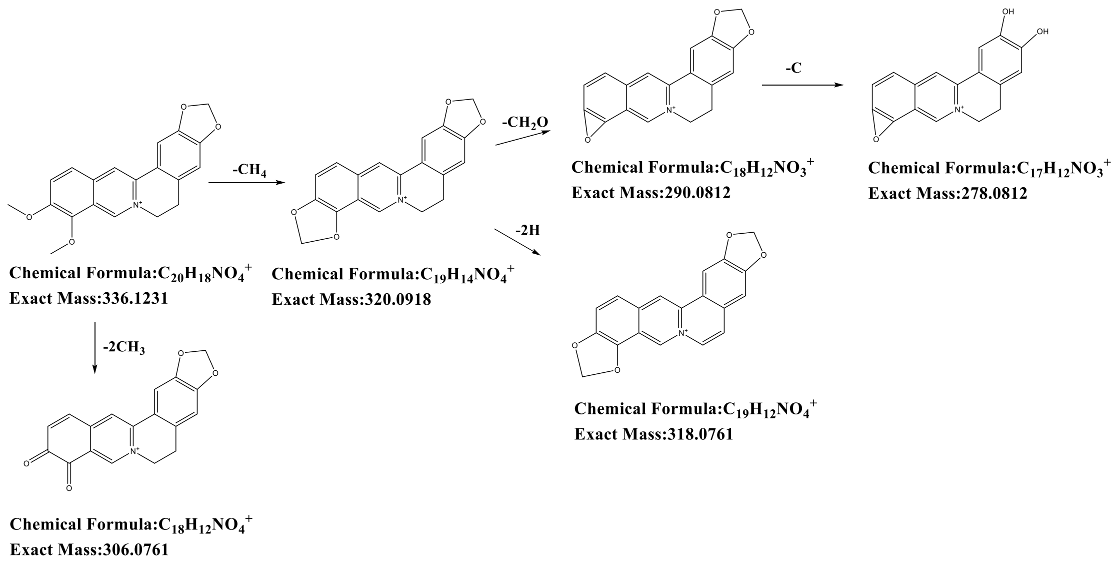

10. Chelerythrine

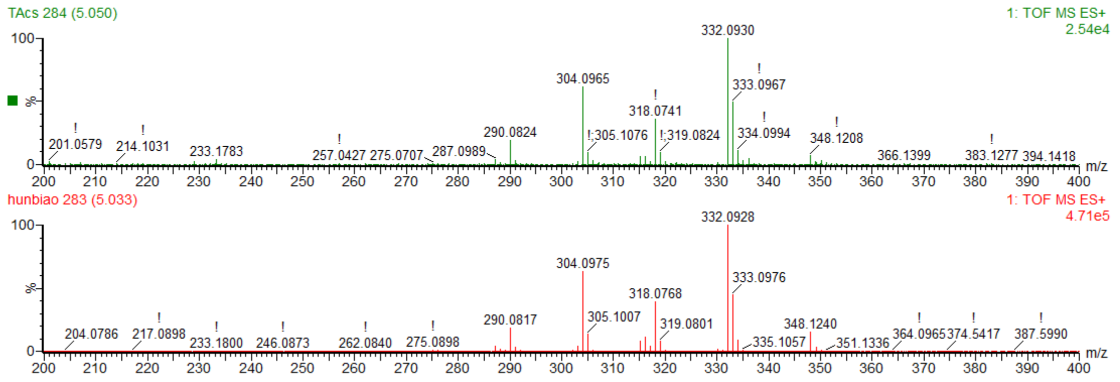

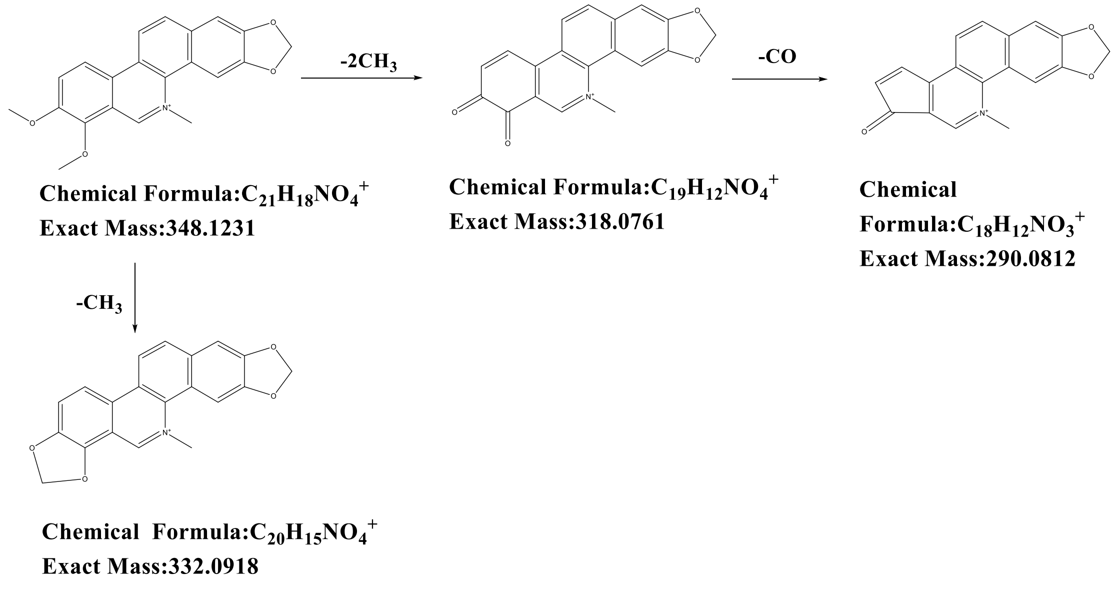

Supplementary Figures

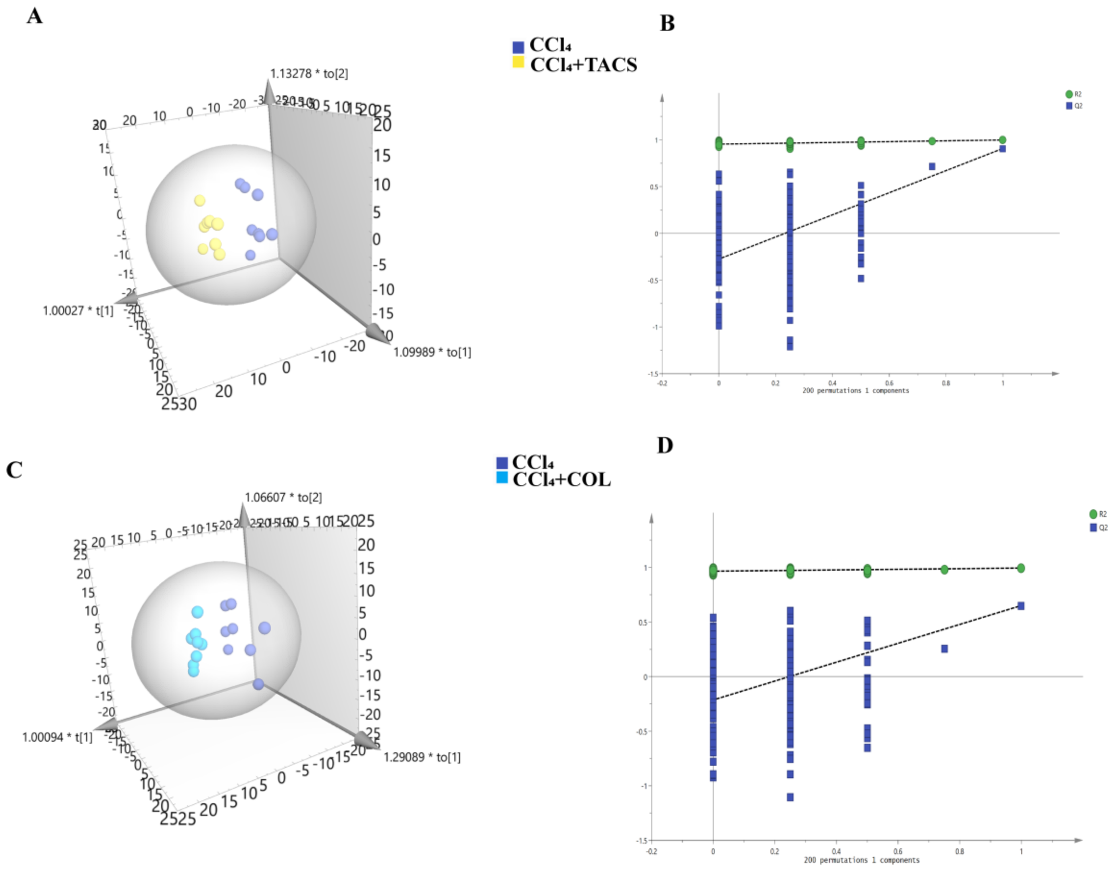

Figure S3. (A, C) OPLS-DA score plot. (A) CCl_4-treated group vs TACS group; (C) CCl_4-treated group vs COL group;(B, D) Permutation test (n = 200 times) was used to validate the OPLS-DA model. (B) CCl_4-treated group vs TACS group;(D) CCl_4-treated group vs COL group.

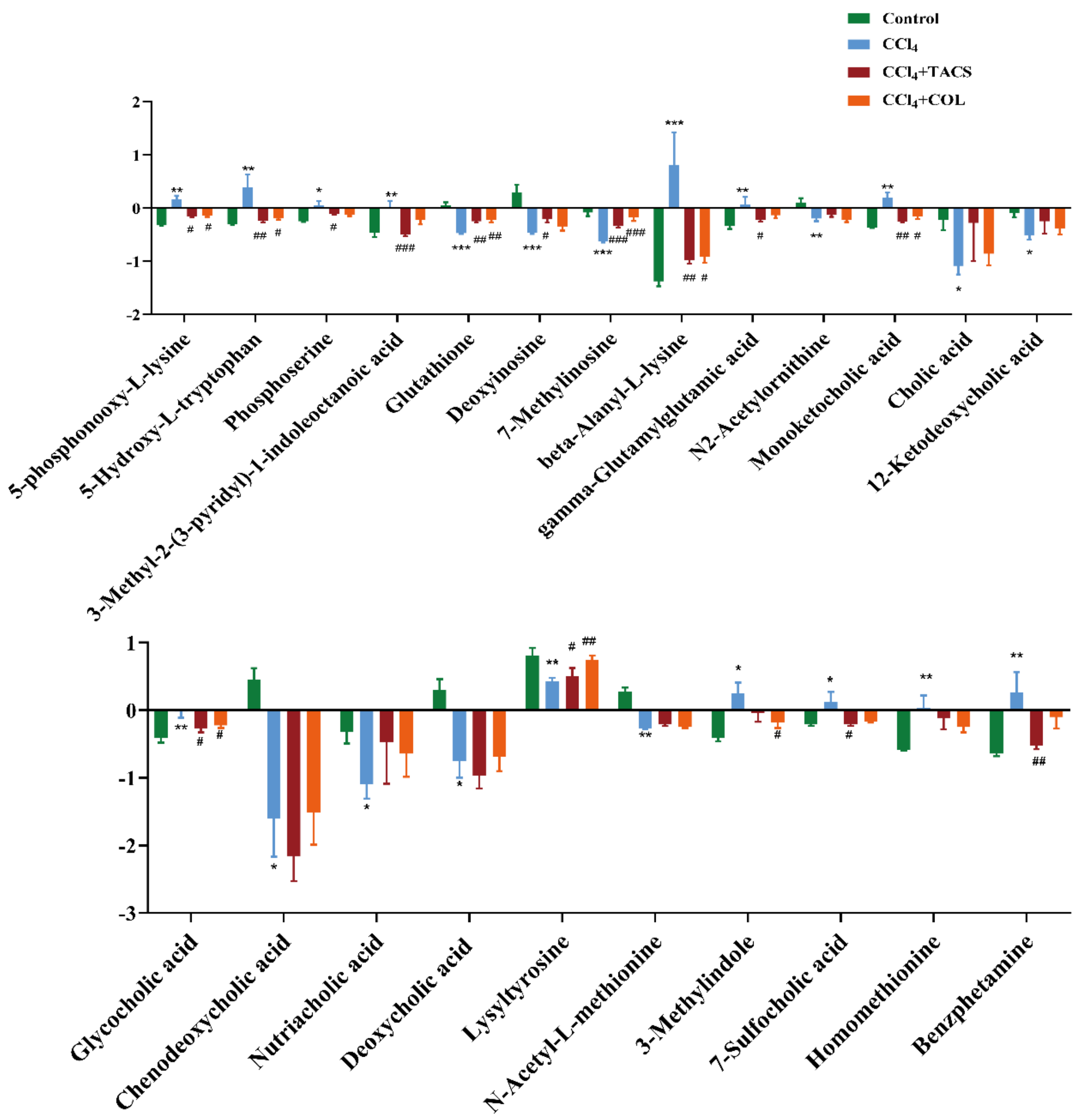

Figure S4. Levels of differential metabolites in feces after intervening with TACS and colchicine (n=8). Data were presented as the mean ± SEM. $^{*}P < 0.05$, $^{**}P < 0.01$, $^{***}P<0.001$ compared with control group; $^{\#}P < 0.05$, $^{\#\#}P < 0.01$, $^{\#\#\#}P < 0.001$ compared with CCl_4-treated group.

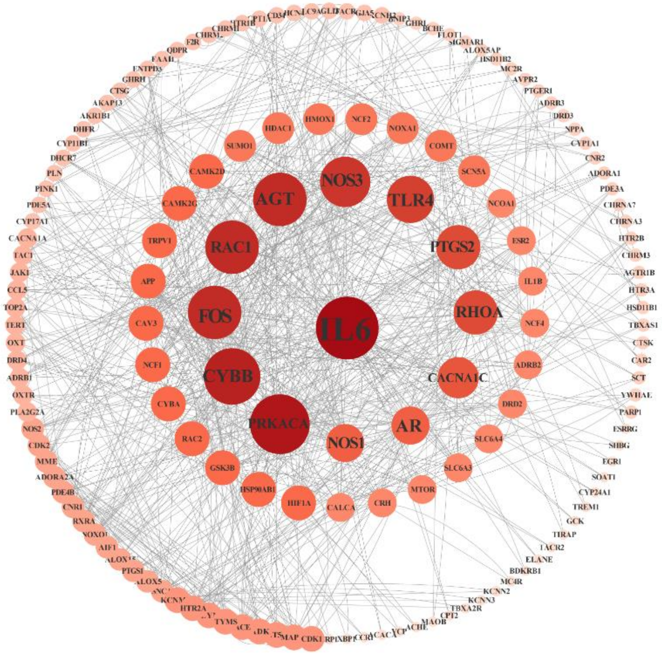

Figure S5. The PPI network of TACS treatment on liver fibrosis. Node color reflects its degree.

Supplementary Tables

Table S2 The reproducibility of UPLC-Q-TOF/MS method validation under the positive ion mode using QC samples

NO	RT(min)	m/z	repeatability	
			$RSD(\%)_{RT}$	$RSD(\%)_{m/z}$
1	0.886	219.0312	0.00000	0.00016
2	1.187	132.1071	0.00000	0.00029
3	2.727	166.0912	0.00000	0.00028
4	5.006	396.8046	0.00000	0.00017
5	6.661	350.1423	0.00000	0.00015
6	12.905	274.2787	0.00342	0.00023
7	14.257	432.2406	0.00370	0.00019
8	17.961	299.2624	0.01842	0.00020

Table S3 The parameters for evaluating the model quality of PCA in positive ion modes.

Groups	Components	$R^2X(cum)$	$Q^2(cum)$
Control vs CCl_4vs TACS vs COL vs QC	3	0.743	0.678

Table S4 The parameters for evaluating the model quality of PLS-DA in positive ion modes.

Groups	Components	R^2X(cum)	R^2Y(cum)	Q^2(cum)
Control vs CCl_4vs TACS vs COL	3	0.34	0.825	0.65

Table S5. The parameters for evaluating the model quality of OPLS-DA in positive ion modes.

Groups	Components	R^2X(cum)	R^2Y(cum)	Q^2(cum)
Control vs CCl_4	3	0.449	0.996	0.893
CCl_4 vs TACS	3	0.36	0.998	0.908
CCl_4+Colchicine	3	0.376	0.993	0.65

Table S6. Levels of differential metabolites in feces(n=8)

Metabolites	Control	CCl_4	TACS	COL
5-phosphonooxy-L-lysine	-0.3218±0.00791	0.1575±0.7429**	-0.1583±0.1891##	-0.1452±0.02309#
5-Hydroxy-L-tryptophan	-0.3049±0.01939	0.3899±0.23697**	-0.2467±0.03242#	-0.1899±0.02802
Phosphoserine	-0.2483±0.01550	0.0443±0.08596*	-0.1150±0.01546#	-0.1219±0.02781
3-Methyl-2-(3-pyridyl)-1-indoleoctanoic acid	-0.4711±0.07640	0.0139±0.11982**	-0.4937±0.03203###	-0.2318±0.7986##
Glutathione	0.0433±0.0623	-0.473±0.01548***	-0.2471±0.02411##	-0.2212±0.04729
Deoxyinosine	0.2955±0.13818	-0.4711±0.01541***	-0.2037±0.07118#	-0.3456±0.07977
7-Methylinosine	-0.0864±0.06604	-0.6284±0.02169***	-0.3317±0.04212###	-0.1722±0.07341###
beta-Alanyl-L-lysine	-0.3780±0.9545	0.8102±0.60543***	-0.9813±0.06928##	-0.9179±0.1097#
gamma-Glutamylglutamic acid	-0.3398±0.05757	0.0662±0.14012**	-0.2471±0.02411#	-0.2212±0.04729
N2-Acetylornithine	0.1031±0.08026	-0.1976±0.05154**	-0.1337±0.3599	-0.2319±0.03350
Monoketocholic acid	-0.3667±0.00844	0.1978±0.9268**	-0.2523±0.2238##	-0.1573±0.04884#
Cholic acid	-0.2464±0.20217	-1.1219±0.17150*	-0.1958±0.83333	-0.8940±0.22281
12-Ketodeoxycholic acid	-0.0907±0.08788	-0.5095±0.08373*	-0.2475±0.23383	-0.3899±0.10681
Glycocholic acid	-0.4064±0.07300	-0.0215±0.09190**	-0.2714±0.05291#	-0.2255±0.03738#
Chenodeoxycholic acid	0.4515±0.17037	-1.6073±0.56277*	-2.1558±0.37578	-1.5179±0.47203
Nutriacholic acid	-0.3196±0.17318	-1.0966±0.2144*	-0.4747±0.61241	-0.6449±0.33956

Deoxycholic acid	0.3012±0.15723	-0.7526±0.24399*	-0.9693±0.18871	-0.6836±0.21802
Lysyltyrosine	0.8057±0.112293	0.4310±0.04725**	0.5062±0.11706#	0.7465±0.06202##
N-Acetyl-L-methionine	0.2714±0.06493	-0.2701±0.00636**	-0.2112±0.2177	-0.2482±0.1684
3-Methylindole	-0.4092±0.05298	0.2448±0.16353	-0.401±0.13057	-0.1781±0.08787
7-Sulfocholic acid	-0.2029±0.02808	0.1253±0.14764*	-0.2078±0.0219#	-0.1675±0.01590
Homomethionine	-0.5868±0.01375	0.0358±0.17922**	-0.1125±0.17324	-0.2499±0.07809
Benzphetamine	-0.6446±0.03523	0.2582±0.30470**	-0.5215±0.05565##	-0.1002±0.17094

Data were presented as the mean ± SEM. *$P < 0.05$, **$P < 0.01$, ***$P<0.001$ compared with control group; #$P < 0.05$, ##$P < 0.01$, ###$P < 0.001$ compared with CCl_4-treated group.

Table S7. The relevant detailed parameters in the pathway enrichment.

Pathway Name	p	-log(p)	Holm p	FDR	Impact
Primary bile acid biosynthesis	0.011244	1.9491	0.94447	0.94447	0.02285
beta-Alanine metabolism	0.20177	0.69514	1	1	0
Lysine degradation	0.23559	0.62785	1	1	0.04695
Glutathione metabolism	0.26006	0.58492	1	1	0.25596
Cysteine and methionine metabolism	0.29923	0.524	1	1	0
Glycine, serine and threonine metabolism	0.30683	0.51311	1	1	0.04254
Fatty acid elongation	0.34366	0.46387	1	1	0.00274
Fatty acid degradation	0.34366	0.46387	1	1	0.00335
Tryptophan metabolism	0.35788	0.44627	1	1	0.13094
Purine metabolism	0.51287	0.28999	1	1	0.00613

Table S8. The core targets of TACS treatment on liver fibrosis (Top 10)

NO	Target	Betweenness Centrality	Closeness Centrality	Degree
1	IL6	0.19115536	0.37408313	18
2	PRKACA	0.14588174	0.35831382	17
3	CYBB	0.05426848	0.32692308	16
4	FOS	0.18792862	0.37777778	15
5	AGT	0.16221345	0.32484076	15
6	RAC1	0.02969988	0.31097561	15
7	NOS3	0.06349847	0.33849558	14
8	TLR4	0.04477566	0.3161157	13
9	RHOA	0.05846001	0.34151786	12
10	PTGS2	0.09448701	0.30661323	12

Table S9. The relevant information of molecular docking

Related pathway	Chemical component	Target protein	PDB ID	Total scores	Conformations	Binding energy(Kcal/mol)
Tryptophan metabolism	Tetrahydropalmatine	CYP1A1	4I8V	3.7236	10	-21.24
	Palmatine			4.3099	19	-24.59
	Epiberberine	MAOB	1O5W	5.2029	14	-29.68
	Jatrorrhizine			4.8005	11	-27.39
	Dehydrocavidine			5.6463	11	-32.21
	Palmatine			4.0848	15	-27.66
	Berberine			3.9206	5	-22.37
	Chelerythrine			3.9483	9	-22.52
Glutathione metabolism	Tetrahydropalmatine	ODC1	1D7K	5.2303	20	-29.84
